# Genome-wide methylation study on depression: differential methylation and variable methylation in monozygotic twins

**DOI:** 10.1038/tp.2015.49

**Published:** 2015-04-28

**Authors:** A Córdova-Palomera, M Fatjó-Vilas, C Gastó, V Navarro, M-O Krebs, L Fañanás

**Affiliations:** 1 Unitat d́Antropologia, Departament de Biologia Animal, Facultat de Biologia and Institut de Biomedicina (IBUB), Universitat de Barcelona, Barcelona, Spain; 2 Centro de Investigaciones Biomédicas en Red de Salud Mental (CIBERSAM), Instituto de Salud Carlos III, Madrid, Spain; 3 Instituto de Investigaciones Biomédicas August Pi i Sunyer (IDIBAPS), Barcelona, Spain; 4 Departamento de Psiquiatría and Instituto Clínico de Neurociencias (ICN), Hospital Clínico, Barcelona, Spain; 5 Université Paris Descartes, PRES Paris Sorbonne Cité, INSERM, Laboratoire de Physiopathologie des Maladies Psychiatriques, Centre de Psychiatrie et Neurosciences, Paris, France; 6 Hôpital Sainte-Anne, Service Hospitalo-Universitaire, Faculté de Médecine Paris Descartes, Paris, France; 7 GDR3557-Institut de Psychiatrie, Paris, France

## Abstract

Depressive disorders have been shown to be highly influenced by environmental pathogenic factors, some of which are believed to exert stress on human brain functioning via epigenetic modifications. Previous genome-wide methylomic studies on depression have suggested that, along with differential DNA methylation, affected co-twins of monozygotic (MZ) pairs have increased DNA methylation variability, probably in line with theories of epigenetic stochasticity. Nevertheless, the potential biological roots of this variability remain largely unexplored. The current study aimed to evaluate whether DNA methylation differences within MZ twin pairs were related to differences in their psychopathological status. Data from the Illumina Infinium HumanMethylation450 Beadchip was used to evaluate peripheral blood DNA methylation of 34 twins (17 MZ pairs). Two analytical strategies were used to identify (a) differentially methylated probes (DMPs) and (b) variably methylated probes (VMPs). Most DMPs were located in genes previously related to neuropsychiatric phenotypes. Remarkably, one of these DMPs (cg01122889) was located in the *WDR26* gene, the DNA sequence of which has been implicated in major depressive disorder from genome-wide association studies. Expression of *WDR26* has also been proposed as a biomarker of depression in human blood. Complementarily, VMPs were located in genes such as *CACNA1C*, *IGF2* and the p38 MAP kinase *MAPK11*, showing enrichment for biological processes such as glucocorticoid signaling. These results expand on previous research to indicate that both differential methylation and differential variability have a role in the etiology and clinical manifestation of depression, and provide clues on specific genomic loci of potential interest in the epigenetics of depression.

## Introduction

Depressive psychopathology has been shown to be highly influenced by environmental factors, some of which are believed to exert stress on human brain functioning via epigenetic modifications.^1, 2, 3^ Accordingly, the search for precise molecular epigenetic signatures underlying this environmental impact is a current trend in the field.

Among several different epigenetic marks, DNA methylation is particularly interesting in this context, as previous evidence indicates that depressed individuals exhibit particular profiles of both methylation levels (that is, hyper- and hypomethylation at some loci) and methylation variance (that is, increased variance in affected subjects).^4, 5, 6^ The number of publications relating DNA methylation to these conditions has been increasing in recent years; overall, they suggest an association, even when typically studying DNA samples from peripheral tissues of unrelated individuals.^6^

Notably, a substantial degree of the DNA methylation profile is determined by the underlying DNA sequence of the organism,^7, 8^ suggesting that some adjustment for inter-individual sequence differences is required when associating this epigenetic mark with other phenotypes. As pairs of monozygotic (MZ) twins have almost identical DNA sequences.^9, 10^ studies of their phenotypic discordance provide a valuable tool in epigenetic research.^11^ Methylation profiles of members of a MZ twin pair may be very similar not only due to their DNA sequence resemblance, but also as a consequence of shared zygotic epigenetic features and a common (pre- and post-natal) environment, among other issues.^8, 11^ Hence, differences in their DNA methylation levels arise in response to unique environmental factors and stochastic influences.^12, 13^

In this sense, a consistent finding in the literature is the increased variance of genome-wide DNA methylation profiles of affected co-twins in depression-discordant pairs.^4, 5, 14^ As intrapair differences in MZ co-twins are related not only to environmental but also to stochastic epigenetic factors,^12, 13^ a feasible hypothesis is that mean DNA methylation level differences—measured as differentially methylated probes (DMPs)—could be linked to environmental factors related to the etiology and clinical manifestation of a disease; complementarily, the changes in methylation variance—measured as variably methylated probes (VMPs)—may be associated with stochasticity.

Although this idea of epigenetic stochasticity has been little explored in relation to psychiatric disorders, research mainly on cancer shows that stochastic epigenetic processes have a dear role in the difference between health and disease phenotypes.^15, 16, 17, 18, 19^ Notably, some methodological tools based on second-moment statistics (that is, variance) of genome-wide DNA methylation profiles have recently been introduced to discriminate between affected and unaffected samples;^20, 21^ their biological significance largely relies on the effects of stochastic epigenetic factors. Broadly speaking, these tools have been developed in recognition that most genomic regions do not exhibit DNA methylation variability and, thus, small numbers of VMPs across the genome are typically identified in complex diseases.^20, 21^ Interestingly, it has recently been proposed that similar analytical approaches may be useful to study depressive psychopathology.^22^

To our knowledge, no previous study has indicated specific genomic loci at which methylation variability may have relevance for psychopathology. This is particularly important in light of the three previous reports showing increased genome-wide methylation variance in depressed MZ co-twins from discordant pairs.^4, 5, 14^

Accordingly, the present study aimed to identify epigenetic differences in depressive psychopathology using two distinct analytical strategies. First, a widely known genome-wide methylation approach that detects DMPs on the basis of both the statistical significance and the magnitude of DNA methylation differences was implemented. This approach has proven to be useful in DNA methylation studies in psychiatry.^5, 23, 24^ Second, the genomic loci of the CpG probes exhibiting DNA methylation variance that could be associated with disease were obtained using an analytic methodology proposed herein; this approach is especially suited to identify VMPs in samples consisting of disease-concordant, discordant and healthy MZ twins. This method assumes that stochastic epigenetic variance among diagnostic-concordant pairs is related to etiological and symptomatic differences within pairs,^25, 26^ whereas epigenetic variability found only in diagnostic-discordant pairs would relate to a more homogeneous core of the disease.

## Materials and methods

### Sample description

Participants in this study were part of a larger ongoing twin sample (UB-Twin Registry) consisting of 242 Caucasian Spanish adult twins from the general population who gave permission to be contacted for research purposes. For that sample, the exclusion criteria included age under 18 and over 65 years, a medical history of neurological disturbance, presence of sensory or motor alterations and current substance misuse or dependence. Written informed consent was obtained from all participants after a detailed description of the study aims and design, as approved by the local Ethics Committee.

Medical records and a battery of psychological and neurocognitive tests were obtained in face-to-face interviews by trained psychologists. In addition, peripheral blood or saliva samples were obtained from all 242 participants. The zygosity of the pairs was determined by genotyping 16 highly polymorphic microsatellite loci from DNA samples (SSRs; PowerPlex 16 System; Promega, Madison, WI, USA). Identity on all the markers can be used to assign monozygosity with greater than 99% accuracy.^27^

A final group of 34 middle-aged participants (17 MZ twin pairs; age range 22–56 years, median age 38 years; 47% female) who were representative and informative for psychopathology, neurocognition and early stress factors was extracted from the above-described sample, to be investigated for brain function and genome-wide epigenetic signatures. Similar MZ twin sample sizes have previously been used in comparable literature reports.^5, 14^ Peripheral blood was available from all members of this group. All analyses described below refer to this 34-individual subset.

### Clinical evaluation

A trained clinical psychologist applied the Structural Clinical Interview for DSM-IV Axis I Disorders (SCID-I)^28^ in a face-to-face interview to screen for the presence of any lifetime depression (F32.x) or related anxiety spectrum disorder (F40.x and F41.x). This apparently broad category of outcomes was used in conjunction with evidence on the comorbidity, shared etiopathology and diagnostic criteria overlap between depressive and anxious disorders,^6, 29, 30, 31, 32^ as well as taking into account evidences of some shared DNA methylation mechanisms in these diagnoses.^6^

Individuals meeting the diagnostic criteria for at least one lifetime diagnosis of (DSM-IV) anxious or depressive disorder were classified as affected by a stress-related disorder, and ‘concordant', ‘discordant' and ‘healthy' statuses of twin pairs were defined accordingly. Specifically, there were seven healthy pairs, six discordant and four concordant pairs for lifetime DSM-IV diagnoses. In addition, on the day of blood sampling, current psychiatric symptoms were evaluated using the Brief Symptom Inventory (BSI).^33, 34^ The BSI is a self-administered 46-item screening instrument aimed at identifying the experience of psychopathological symptoms during the last 30 days. It is composed by six subscales (depression, phobic anxiety, paranoid ideation, obsession-compulsion, somatization and hostility) conceived for use in both clinical and non-clinical samples. Items are rated on a five-point scale of distress, according to self-perception of symptoms. Descriptive data from the current sample are summarized in Table 1.

Overall, there were four concordant, six discordant and seven healthy MZ twin pairs (Table 1). Briefly, in the eight diagnostic-concordant-participant subset (four pairs), there were four individuals with depression (F32.x) and four diagnoses of anxiety disorders (F40.x and F41.x); half of these diagnoses were experienced by the individuals at the moment of blood extraction, and the rest of the individuals had met diagnostic criteria for psychopathology some years before that date (estimated mean (s.d.) elapsed time since last remission: 13.8 (9.2) years; a right-tailed skewed distribution ranging from 2 to 28 years). Regarding the six affected co-twins from the diagnostic-discordant pairs, there were four depression (F32.x) and two anxiety (F41.0) diagnoses; one of them fulfilled diagnostic criteria at the moment of peripheral blood sampling, and the rest of them had met such criteria before. Importantly, despite the apparent clinical heterogeneity, there were statistically significant intergroup differences in the current psychopathological assessment (that is, last-month symptoms measured by the BSI), at the level of both general symptomatology (total BSI score: *P*=0.013) and ongoing depression (depressive BSI subscale: *P*=0.04). Namely, twins with no lifetime history of DSM-IV diagnosis had lower BSI scores—fewer self-reported symptoms—in both the depressive subscale and the whole questionnaire than diagnostic-discordant pairs, whereas the diagnostic-concordant twins had greater BSI scores than discordant and healthy twin groups. Likewise, a logistic regression model was performed to evaluate the relationship between (current) BSI depressive scores and categorical (DSM-IV) diagnoses in the 34-twin sample. After adjusting for the correlated nature of twin data (heteroskedasticity), higher (current depression) BSI scores predicted a greater risk of a clinical diagnosis (*β*=0.362, *P*=0.013, *R*^2^=0.295). Similarly, in the six diagnostic-discordant pairs, the DSM-IV-affected co-twins had higher BSI scores than their healthy co-twins (mean/median score in affected: 4.2/4.5; mean/median score in healthy: 2.8/2.5); however, due to some properties of this small data set, the nonparametric Wilcoxon–Mann–Whitney test could not estimate exact *P*-values due to ties and zeroes.

All participants were asked to report if they had received pharmacological or psychological treatment or had consulted a psychiatrist or psychologist since they first participated in the study. Only one of the 34 participants had lifetime exposure to drug treatment for anxiety or depression by the time of this study.

### Methylation data

The Illumina Infinium HumanMethylation450 (450 K) BeadChip^35, 36^ was employed with peripheral blood DNA samples for all 34 participants. Specifically, by genotyping sodium bisulfite-treated DNA, DNA methylation is assayed by this platform at >450,000 CpG sites across the genome at single-base resolution; next, bisulfite-converted DNA undergoes whole-genome amplification, before being fragmented and hybridized to microarray probes. The DNA methylation fraction of each CpG site is estimated as *β*=*M*/(*M*+*U*+*α*); *M* and *U* stand for methylated and unmethylated fluorescence intensities, and *α* is an arbitrary offset applied to stabilize *β*-values with low intensities.

### Statistical analyses

#### DMPs

In order to find DMPs, a previously described analytical approach^5, 23, 24^ was conducted using, initially, data from the six depression-discordant twin pairs. This method aims to rank all CpG probes in the array. The highest-ranked probes are those with a combination of low *P*-value and high mean difference (Δ*β*). Briefly, the first step consists in conducting a paired *t*-test for every CpG probe in the array; a score is assigned to every probe depending on its *P*-value: the lower the *P*-value, the higher the score. Afterward, the absolute mean intrapair difference is estimated for each CpG probe, and a second score is assigned to every probe: the larger the methylation difference (absolute Δ*β*), the higher the score. The two scores are combined (that is, added) for every probe, and all probes are ranked from high to low scoring. Namely, probes with both a low *P*-value and a relatively large methylation difference are in the top of the rank. From this general rank for the >450,000 probes, a list of the top 10 probes (that is, those with the best arrangements of low *P*-value and high Δ*β*) was extracted.

To further validate these CpG sites, an additional step was undertaken using the information from diagnostic-concordant and healthy pairs. The mean absolute differences (|Δ*β*|) in DNA methylation were retrieved for the three groups to test the null hypotheses that these top 10 CpG probes found in the discordant co-twins that have also large methylation differences in either concordant and healthy pairs. This additional test allowed assessing whether the top 10 DMP probes often display methylation differences within MZ pairs, regardless of their phenotypic statuses. Large intrapair methylation differences across all pairs would indicate that a given CpG site may be environmentally sensitive, but not linked to the etiopathology of depression. Thus, by performing Wilcoxon–Mann–Whitney tests, it was evaluated whether DNA methylation differences within discordant pairs are larger than those found in either concordant or healthy pairs (|Δ*β*_discordant_|>|Δ*β*_concordant_| and |Δ*β*_discordant_|>|Δ*β*_healthy_|). Additional information about this procedure can be found in Supplementary Table 1.

#### VMPs

A data-driven analytical approach using information from all concordant, discordant and healthy MZ twins was used. Initially, absolute intrapair differences in DNA methylation levels were computed for all >450,000 CpG sites across the genome for all 17 MZ twin pairs. From this information, the median value of absolute DNA methylation (intrapair) differences is computed for each diagnostic group (concordant, discordant and healthy) at each of the >450,000 probes. These median values are used as centrality measures since they are more robust to outliers than conventional arithmetic means.

After this step, an *m* × 3 matrix is retrieved, where *m* stands for the number of CpG sites considered (>450,000) and 3 is the number of diagnostic groups (here, concordant, discordant and healthy). Each cell contains the median value of the intrapair absolute methylation difference observed for a given diagnostic group at a specific CpG site.

Note that information introduced to the previous matrix does not contain any clue about direction of the differences. Some assumptions are used to further interpret this information: (i) probes with large intrapair methylation differences across only discordant co-twins—that is, with no intrapair differences in concordant and healthy twins—could have arisen from stochastic factors altering the methylation level of the affected co-twins' (in discordant pairs); due to this stochasticity, the affected co-twin may have transitioned from the normal methylation level of his/her healthy co-twin to either hyper- or hypomethylation. (ii) CpG sites with large methylation differences only in diagnostic-concordant MZ pairs should be linked to symptom heterogeneity of a pair^26, 37^ and (iii) probes with large intrapair differences only in healthy pairs should be relatively less frequent—methylation stochasticity is typically associated with disease—^15–18^ and may index processes that are either not related to the specific physiopathological conditions studied here, or normally activated in health (but dynamically constrained in disease).

The next step consists in determining a methylation difference threshold above which the observed variability for each diagnostic group and at each CpG site (the median value of the absolute intrapair differences) can be considered large. As previous reports indicate that methylation differences above 10% in Illumina assays have biological significance and have a low probability of being technical artifacts,^38, 39, 40^ a CpG site was considered ‘variable' if above this Δ*β*≥|0.1| threshold. Of note, as shown in the Results section, CpG probes with an intrapair Δ*β*≥|0.1| are highly infrequent in this data set, suggesting this may be a proper threshold.

Then, after the previous step, all >450,000 CpG sites are examined to determine which of them show variability only within concordant, discordant or healthy pairs; they are later examined via pathway analysis to evaluate the potential biological relevance of their stochastic disruptions.

#### Pathway analysis

Relevant sets of genes from whole-genome analyses indicated in the Results section were uploaded to Cytoscape (version 3.0.2)^41^, using Reactome FI Cytoscape Plugin 4,^42^ network version 2013, to obtain data on underlying reactions, pathways and biological processes.

All the outcome assessment steps (that is, statistical analyses) described in this section were conducted by an investigator (AC-P) who did not participate in sample collection, clinical evaluation, zygosity determination or DNA methylation measurement and data pre-processing.

## Results

### DMPs

With data from the six diagnostic-discordant MZ pairs, an analytic approach previously reported in Psychiatric Epigenetics studies^5, 23, 24^ was used to identify the top 10 CpG sites with the largest methylation differences and smallest *P*-values. Table 2 contains information on these 10 CpG sites across the genome. Δ*β*- and *P*-values were in the range of those reported previously in similar study designs.^5^ Remarkably, as shown in Table 2, most probes were located in genes previously reported in neuropsychiatric studies.

Of particular interest in this 10-site list are cg11433980, cg17798944, cg00567749 and cg01122889. They are respectively located in *CBR3* (carbonyl reductase 3), *RPL3* (ribosomal protein L3), *VCAN* (versican) and *WDR26* (WD repeat domain 26). These genes have previously been associated with depressive phenotypes.^43, 44, 45, 46^ Further information on this finding can be found in the Discussion.

As indicated by the superindices of the probe names (first column of Table 2), neither concordant nor healthy twin pairs displayed intrapair differences in four out of the initial 10-site list: cg06493080 (*HOXB7*), cg18974921, cg14747903 (*LSR*) and cg01122889 (*WDR26*). This indicates greater robustness of the findings for these four CpG sites than for the other six probes. Namely, in this twin sample, these four CpG sites appeared to be modulated by the environment only in depression-discordant MZ pairs; co-twins from neither concordant nor healthy pairs differed in their methylation levels.

### VMPs

As a whole, DNA methylation profiles were highly correlated across twin pairs, regardless of the diagnostic status of co-twins. A detailed description of these correlations can be found in Supplementary Table 2.

**Figure 1:**
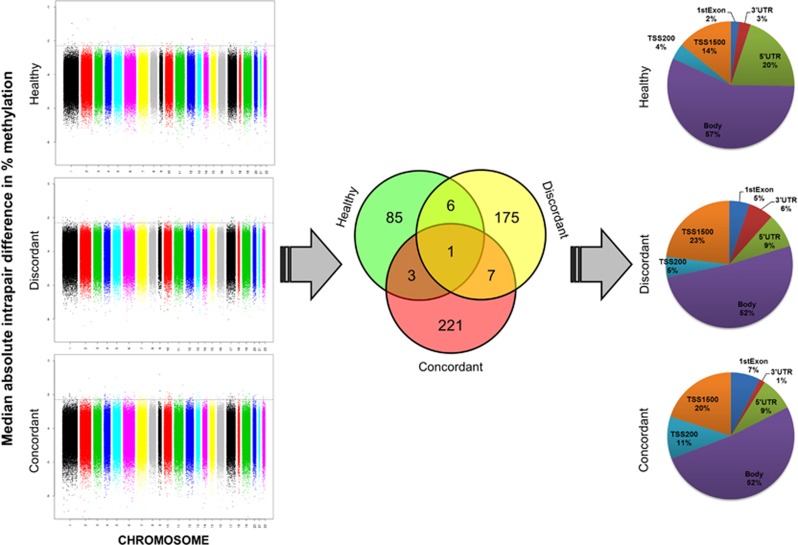
Selection of highly variable CpG sites across DSM-IV diagnostic groups based on large intrapair differences in whole-genome percentage methylation. Left**:** after estimating absolute intrapair differences for each CpG region in every twin pair, median values of these differences were calculated across diagnostic groups and are plotted in a logarithmic scale, according to genomic location. Individual CpGs (dots) above the 10% threshold (dashed line, at log(0.1)=−2.3) methylation differences were identified and separated for the next step. Centre**:** Venn diagram showing intersections and disjunctions of the highly variable CpG probes obtained before. Right**:** gene region feature categories (UCSC) of the CpG sites showing large intrapair differences only in each of the diagnostic groups. Inset numbers represent percentage of CpG sites. 1st exon, first exon; 3′UTR, 3′ untranslated region; 5′UTR, 5′ untranslated region; TSS, transcription start site; TSS1500, within 1.5 kb of a TSS; TSS200, within 200 bps of a TSS.

Briefly, based on previous reports,^38, 39, 40^ a median absolute intrapair difference ≥10% within each twin group (concordant, discordant or healthy) was chosen in this study as a threshold to determine which CpG probes could be considered variable. Only 1.7% of the whole data set of absolute intrapair differences (17 intrapair differences at 485,512 CpG sites: a matrix with 8,055,688 cells) showed values equal to or larger than 10% of the total methylation fraction. In recognition that large intrapair differences are likely to have major biological relevance, the next step consisted of the identification of probes with variability only between each twin set (concordant, discordant or healthy) (Figure 1).

Each of the three diagnostic groups (concordant, discordant and healthy controls) had a specific set of CpG probes with large intrapair differences in methylation fraction; specifically, healthy pairs showed variability at 85 CpG sites that were not variable in the other groups; similarly, discordant and concordant pairs had, respectively, 175 and 221 variable probes that did not show variability in the other diagnostic groups of twins (Figure 1). Of note, healthy co-twins had the least variable genome-wide DNA methylation profile, seemingly in agreement with findings of increased variability in disease.^4, 5, 14^ The distribution of gene region feature categories (UCSC) of each of the three sets of CpG sites was very similar for all three groups (concordant, discordant and healthy). Identifiers and additional data for these individual CpG sites are available in Supplementary Table 3. Owing to the design of the DNA methylation array employed here, even genes with the largest numbers of CpG probes would not be likely to appear by randomly sampling 85, 175 or 221 probes. Further information and discussion about these sampling probabilities can be found in Supplementary Table 4.

**Figure 2:**
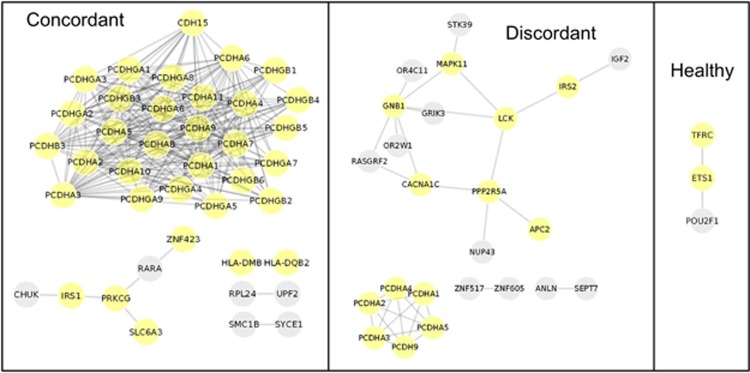
Molecular interaction networks from the lists of genes showing large intrapair differences between each of the diagnostic groups. Left: concordant pairs; center: discordant pairs; right: healthy pairs. Proteins enriched for biological pathways (Table 3) are highlighted in the network diagrams.

Interestingly, pathway analysis using the lists of genes with highly variable probes (from variable CpG sites exclusive of concordant, discordant or healthy twin pairs) generated relatively large functional interaction networks (>10 interacting proteins) only in discordant and concordant pairs (Figure 2). Accordingly, gene lists from the concordant and discordant groups showed enrichment for several processes, some of which are of relevance in neuropsychiatry (Figure 2 and Table 3). For instance, processes such as ‘rapid glucocorticoid signaling', ‘dopaminergic synapse' and ‘interleukin-2-mediated signaling events' were enriched in the VMPs of discordant pairs, whereas ‘nervous system development' was enriched in both concordant and discordant groups. In contrast, the epigenetic variability of the healthy pairs was enriched only for ‘HIF-1-alpha transcription factor network' (Table 3). A detailed list of the VMPs and the full gene lists from which these networks were generated can be found in Supplementary Table 3.

## Discussion

This work evaluated the potential relationship between DNA methylation levels and depressive psychopathology using genome-wide data. Two distinct approaches, each of which has a particular biological meaning, were used: (i) differential methylation (DMPs) and (ii) variable methylation (VMPs). The current design, including healthy, concordant and discordant MZ twin pairs, allowed us to search for potential environmentally induced and pathology-specific DNA methylation differences. Previous reports had shown increased variability in the DNA methylation profiles of affected MZ co-twins from depression-discordant pairs.^4, 14, 23^ However, to the best of our knowledge, this is the first study aimed at unraveling specific genomic loci at which methylation variability may be a marker of depression.

### DMPs

One of the most relevant outcomes of the current study is the association between hypomethylation of cg01122889 in *WDR26* and a lifetime diagnosis of depression. Notably, neither healthy nor diagnostic-concordant MZ pairs exhibited intrapair differences in DNA methylation of this CpG site, indicating that it could be a marker of environmental influences leading to depression. Remarkably, one of the largest meta-analytic studies conducted to date of major depressive disorder (MDD) genome-wide association study data suggests a role for *WDR26*'s rs11579964 single-nucleotide polymorphism—about 80 kbp from cg01122889—in the causality of depression.^45^ Of note, a mega-analysis of MDD genome-wide association study data also reported this and other single-nucleotide polymorphisms close to *WDR26* (rs2088619: Chr1:222825183) as probably predisposing for MDD, although confirmation is needed.^47^ In addition, there is evidence suggesting an association between decreased blood transcript levels of *WDR26* and depression-related phenotypes.^43^ Although using DNA methylation data to derive conclusions about gene expression could be somehow speculative, hypomethylation of gene bodies may be related to lower gene expression.^48, 49^ Accordingly, as cg01122889 is located in *WDR26*'s gene body, its hypomethylation in depressed individuals may be associated with lower gene expression levels, consistent with the findings of Pajer *et al.*^43^ Nevertheless, confirmation is needed, as there is evidence of distinct relationships between *WDR26*'s body DNA methylation and expression (that is, hypomethylation can be associated with either lower or greater gene expression) across a number of distinct healthy and pathological tissues.^50^

Other DMPs found herein were located in genes previously related to depressive phenotypes, such as *CBR3,* *RPL3* and *VCAN*.^43, 44, 46^ For instance, hippocampal upregulation of CBR3 enzymes has been found after antidepressant treatment in adult rats.^44^ As with *WDR26*, the current association between hypomethylation of the *CBR3*'s gene body in adult depressed individuals may be related to lower expression of CBR3, which may be compensated by antidepressant treatment. A similar argument could be proposed for *VCAN*: the hypomethylation in depressed individuals of the present sample somehow resembles the decreased gene expression in an animal model of depression proposed by Pajer *et al.*^43^ Note that current results for both *VCAN* and *WDR26* are consistent with those proposed by Pajer *et al.*^43^ Likewise, Lee *et al.*^46^ reported downregulation of *RPL3* hippocampal gene expression in an animal model of stress, in agreement with the current DNA methylation finding.

The above results were obtained by following a sound methodological procedure.^4, 5, 23^ However, it is worth noting that, along with *WDR26*'s cg01122889, methylation changes in cg06493080 (*HOXB7*), cg18974921 and cg14747903 (*LSR*) seem to be cross-validated by comparison of the diagnostic-discordant group of twins with concordant and healthy pairs. This follows from the observation that diagnostic-concordant and healthy co-twins have very similar methylation levels at these four sites. Namely, probably only through a differential environmental influence (which would have taken place in affected co-twins from discordant pairs) may a co-twin differ from his/her healthy counterpart. cg06493080 is located in *HOXB7*, a target of the neurodevelopmental gene *FOXP2*. A different functional role has been described for *LSR*, which may predispose to neurocognitive disorders.

### VMPs

As MZ twins are very similar at the epigenome-wide level,^8^ they offer an opportunity to search for markers of stochasticity. Accordingly, an analytic approach was undertaken to determine whether epigenetic instability—as indexed by large and non-systematic DNA methylation differences—within MZ pairs could be related to depressive psychopathology.

In this sample, healthy co-twins had less variable probes than concordant and discordant pairs. It was found that highly variable probes in depression-discordant MZ pairs were located in genes within enriched biological pathways and previously associated with depression, such as *CACNA1C*, *IGF2* and *MAPK11* (Figure 2 and Table 3; additional information in Supplementary Table 3).

In this respect, DNA sequence variation of *CACNA1C* has been widely recognized as a susceptibility factor for depressive psychopathology,^45^ and methylation changes of *CACNA1C* have likewise been associated with early-life stress,^51, 52^ a risk factor for depressive disorders.^53^ Similarly, depressive behavior is likely modulated by *IGF2*.^54, 55^ Also, MAPK11 is one of the four p38 mitogen-activated protein kinases, the activity of which has been linked to depression and related phenotypes.^56, 57^

It is also worth mentioning some findings from the VMP analysis in diagnostic-concordant twin pairs, as one could speculate that stochastic variability within these MZ twins may correlate with etiological and symptomatic heterogeneity. Namely, even though depression-concordant MZ pairs show considerable symptom similarity,^25^ previous research has shown an important role for unique environmental differences in fostering psychopathological heterogeneity within these MZ twin pairs.^58, 59^ Thus, it is also worth noting that genes with large DNA methylation intrapair differences across diagnostic-concordant pairs were enriched for ‘nervous system development', by the interaction of protocadherin members and ZNF423 (Table 3 and Figure 2). Protocadherin encodes a family of proteins that are expressed in neurons and are relevant in synaptic functions, and whose DNA methylation may be altered in response to early-life stress;^60, 61, 62, 63^ the *ZNF423* gene has been associated with the cerebellum-related Joubert syndrome.^64^ Also, the dopamine transporter gene *SLC6A3*, whose DNA sequence and methylation levels have been found to correlate with depressive psychopathology,^65, 66, 67^ was within the enriched pathways (Table 3).

The current findings, which are indicative of stochastic variation in depression, are particularly important in view of recent publications from Feinberg *et al.*^68, 69^ They postulated the existence of variably methylated regions as a mechanism to explain developmentally regulated epigenetic plasticity; they have likewise provided evidence of stochastic DNA methylation variation across a population, even when analyzing samples from the same tissue and the same DNA sequence (that is, isogenic mice). These findings in genetically identical individuals (MZ twins) are in line with their reports, as they also described the abundance of variably methylated regions at loci important for neural and immune system development.

Importantly, a novel data-driven strategy was employed here to detect VMPs. Although other statistically sound tools to compare methylation variance between healthy and affected population samples have recently been introduced;^20, 70^ the present strategy is particularly suited for samples consisting of concordant, discordant and healthy MZ twin pairs. It is worth mentioning that the potential biological significance of the VMPs detected here—as suggested by the pathway analysis—confirms the feasibility of this analytical approach to provide new insights about biological mechanisms underlying some pathologies.

### Overlap in findings from DMPs and VMPs

The fact that no top DMP was found within a gene exhibiting variable methylation is consistent with the notion that different epigenetic mechanisms influence DMPs and VMPs.^68, 69^ However, some interplay may exist between these forms of epigenetic regulation. For instance, it is worth noting that WDR26 (encoded by a gene containing a DMP) suppresses the MAPK signaling pathway,^71^ and that a VMP was found in *MAPK11*. As a member of the p38 family, MAPK11 may participate in the stress response,^72^ and was found here to be enriched within pathways such as ‘glucocorticoid signaling' and ‘interleukin-2-mediated signaling', both of which are likely related to depressive psychopathology. Importantly, this suggestion is derived from data from diagnostic-discordant pairs.

Hence, the current findings may be indicative of the interplay between different epigenetic regulation mechanisms, and suggest that the combination of distinct differentially and variably methylated loci may have an important role in the biological signatures of depression.

### Limitations of the study

Three main limitations of this exploratory study deserve consideration. Namely, the generalizability of the present findings may be limited by (i) the sample size employed, (ii) the clinical heterogeneity of the individuals and (iii) the lack of confirmatory DNA methylation analysis using bisulfite pyrosequencing.

These limitations should be understood in the wider context of the current study design. First, the sample size is small. Although replication using larger independent samples is definitely required, it is worth noting that the current findings are consistent with previous literature on the biological mechanisms underlying depression, probably suggesting the presence of strong effect sizes for the above mentioned relationships between methylation and depression (Table 2 and Table 3). Likewise, the use of three different groups of twin pairs (concordant, discordant and healthy) allowed contrasting some putative epigenetic signatures of environmental stress in diagnostic-discordant co-twins with corresponding measurements in concordant and healthy pairs, which has rarely been done in former studies. As shown in Table 2 and Table 3, the comparison of epigenetic signatures in discordant twins with the methylation profiles of healthy and concordant pairs added robustness to some findings on both DMPs and VMPs.

Another potential limitation of this work is the phenotypical (that is, clinical) diversity of the participants. Although comparing the present results with independent data sets from individuals with a narrower phenotypic distribution (that is, a more clinically homogeneous population) would definitely be useful, two features of the present sample should be noted. First, although there were some individuals with predominantly anxious psychopathology, Table 1 shows that, as a group, concordant co-twins have greater scores in depressive symptomatology during the last month than discordant pairs, and that discordant pairs had more (current) depressive psychopathology than healthy controls. Thus, in contrast to the apparent diagnostic heterogeneity of the participants, the sample has an acceptable distribution with regard to current depression. It is important mentioning that this fact implies that the associations found here can probably be interpreted only in relation to depressive symptoms but not as linked to early etiological mechanisms.

Similarly, as noticed in the Clinical evaluation subsection, it is worth observing that previous research on the epidemiological features and the epigenetic signatures of internalizing disorders (that is, depression and anxiety) indicate important overlaps in etiopathology, diagnostic criteria and DNA methylation profiles across these diagnoses.^6, 29, 30, 31, 32^ Of note, these overlaps may have allowed detecting epigenetically altered genes and pathways with biological relevance for depression.

An additional limitation of this work is the lack of confirmatory DNA methylation analysis by techniques such as bisulfite pyrosequencing. However, it should be noted that previous technical research has pointed out that the sensitivity of Illumina DNA methylation microarrays increases with *β*-value differences.^73^ Accordingly, it has been suggested that, on average, values of Δ*β*≥0.136 can be detected with 95% confidence. Although the methylation assessment conducted here would definitely be more robust if confirmed by bisulfite pyrosequencing, having used methodologies focused on relatively large methylation differences—for both DMPs and VMPs—adds some soundness to the findings.

### Additional implications and future directions

Some points raised by this study deserve further discussion. First, it is important recalling that a common assumption of several psychiatric epigenetic studies is that DNA methylation of blood and brain may correlate and explain variability in psychopathology.^74^ Although the work from several independent laboratories has demonstrated broad cross-tissue differences in methylation profiles,^75, 76, 77, 78^ there is also evidence of a correlation between DNA methylation levels across peripheral lymphocytes and a number of brain regions in response to environmental events.^79, 80, 81^ Likewise, similar cross-tissue DNA methylation profiles—at specific genomic loci—have been found in independent samples of individuals with psychopathology.^5^

To the best of our knowledge, this is the fourth study using an epigenome-wide approach in MZ twins to evaluate the relationship between environmentally induced DNA methylation changes and depressive psychopathology. Regarding VMPs, the former three reports^4, 5, 14^ consistently found increased DNA methylation variance in the affected co-twins of discordant pairs. Although they evaluated the overall statistical variance of the methylomic profiles, the present study expands on this topic by assessing which specific genomic loci could underlie this statistical feature. Of note, the data-driven analytical approach developed here to detect VMPs is specifically suited for samples including diagnostic-concordant and healthy pairs, as it is based on contrasting their epigenetic variability with that of discordant co-twins. It is important noting that, due to the novelty of the topic, some statistical protocols to perform genome-wide VMP assessment have just recently been introduced in cancer research^20, 21, 70^ and thus may need additional adjustments for psychiatric phenotypes. This is particularly important in light of recent findings highlighting specific statistical properties of the genome-wide DNA methylation profiles in depression,^22^ which may certainly differ from cancer data sets. Overall, the current biologically plausible findings suggest that the adopted strategy may have statistical and conceptual feasibility.

As regards to DMPs, the above mentioned previous reports have suggested that methylation of different genomic loci may be associated with depression. Namely, there is no unanimous agreement between these studies, probably due to the different clinical and demographic composition of the samples, as well as to the DNA methylation assessment techniques used. For instance, Byrne *et al.*^14^ analyzed *P*-values obtained from the Infinium HumanMethylation450 Beadchip; in contrast, the assessment of potentially DMPs was based on another statistical approach in this and two other studies.^4, 5^ Likewise, inter-study differences between the present work and the report by Dempster *et al.*^5^ may have arisen from clinical and demographic sample differences, as well as from the use of distinct biological tissues (they analyzed saliva DNA samples from severely depressed adolescents). Also, the *ZBTB20* gene-coding region reported by Davies *et al.*^4^ (Chr3:114618751-114619251) is not evaluated by the Illumina assay employed here, and there may also be a lack of statistical power in the present sample.

From the above mentioned observations, some suggestions for further research of DNA methylation in depression can be derived: (i) cross-sample validations in larger MZ twin samples is needed; (ii) selecting the best informative peripheral tissue (probably either blood or saliva) may allow clearer findings; and (iii) finding proper statistical protocols for epigenetic analyses in depression, especially for the examination of VMPs, could likewise allow research advances (despite relatively small samples, the available literature suggests a role for methylation variability in depression).

## Figures and Tables

**Table 1:** Demographic, psychopathological and neurocognitive data for DSM-IV diagnostic concordant, discordant and healthy MZ twin pairs

	*Concordant (8 subjects, 8 females)*		*Discordant (12 subjects, 4 females)*		*Healthy (14 subjects, 4 females)*		*Group comparison*
	*Mean (s.d.)*	*Range*	*Mean (s.d.)*	*Range*	*Mean (s.d.)*	*Range*	*X^2^;*^a^ P
Age	42.5 (13)	22–54	37 (10.9)	20–50	30.3 (7.3)	19–39	5.9; 0.052
IQ	105.1 (12.5)	87–127	108.1 (11.8)	87–131	110.5 (5.5)	103–118	1.9; 0.393
Current psychopathology (total BSI)	27.9 (16.5)	6–57	20.9 (13.3)	4–45	10.6 (9.3)	1–33	8.7; 0.013^b^
Current depressive symptoms (BSI subscale)	6.9 (6.5)	1–20	3.5 (2.7)	0–9	1.7 (1.8)	0–6	6.4; 0.04^b^

Abbreviations: BSI, Brief Symptom Inventory; IQ, intellectual quotient; MZ, monozygotic.

a Kruskal–Wallis *X*^2^, as these variables were continuous.

b Statistically significant *P*-value.

**Table 2:** Top-ranked differentially methylated probes (DMPs) in six adult MZ twin pairs discordant for depression, and potential neuropsychiatric relevance of their associated genes

*Probe name (target ID)*^a^	P*-value*	*Δβ*	*Coordinates (hg19)*	*Gene name (UCSC)*	*Gene region feature category (UCSC)*	*Potential relevance of the gene in neuropsychiatric disorders*
cg06493080b,^c^	0.000574	−0.085	Chr17: 46688310	HOXB7; HOXB7	5′UTR; 1st exon	Target of *FOXP2,* a gene linked to neurodevelopment.^82^ *HOXB7* may diminish cancer tumor risk in schizophrenia.^83^
cg00567749^b^	0.000818	−0.105	Chr5: 82767908	VCAN; VCAN; VCAN; VCAN	5′UTR; 5′UTR; 5′UTR; 5′UTR	Prospective blood transcriptomic marker for depression.^43^ Axonal growth.^84, 85, 86^ Potential genetic overlap between herpes simplex and depression.^87^ Altered expression in the olfactory epithelium in schizophrenia.^88^
cg18974921b,^c^	0.000565	−0.076	Chr11: 78131895	—	—	—
cg14747903b,^c^	0.000571	−0.072	Chr19: 35740509	LSR; LSR; LSR	Body; body; body	Probable role in prosopagnosia and visual agnosia.^d^
cg15696634	0.000682	−0.072	Chr18: 19746953	—	—	—
cg11433980^b^	0.001425	−0.075	Chr21: 37510727	CBR3	Body	Altered hippocampal gene expression by antidepressant treatment in adult rats.^44^
cg01122889b,^c^	0.000806	−0.071	Chr1: 224620779	WDR26; WDR26	Body; body	GWAS hit in major depressive disorder (meta-analysis of three independent samples).^45^ Prospective blood transcriptomic marker for depression.^43^
cg10550693^b^	0.001554	−0.073	Chr11: 64902189	SYVN1; SYVN1	TSS200; TSS200	Potential role in autism.^89, 90^
cg23004466^b^	0.000854	−0.071	Chr7: 106815478	HBP1	5′UTR	Epigenetics of neurodegeneration and Alzheimer's disease.^91, 92, 93, 94^
cg17798944^b^	0.001679	−0.072	Chr22: 39715225	SNORD43; RPL3; RPL3	TSS200; body; body	Hypothalamic–pituitary–adrenal regulation of stress response.^46^ Potential role in the glycobiology of schizophrenia.^95^

Abbreviations: 1st exon, first exon; MZ, monozygotic; TSS, transcription start site; TSS200, within 200 bp of a TSS; 5′UTR, 5′ untranslated region.

Target ID, Illumina identifier; body, within gene body; Δ*β*, mean methylation fraction difference in discordant pairs (co-twin with depression minus co-twin without depression).

a Superindices next to each probe name indicate whether or not there were (absolute) methylation differences in concordant and healthy pairs.

b Absolute intrapair differences in the four diagnostic-concordant MZ pairs were significantly smaller than in the discordant pairs.

c Absolute intrapair differences in the seven healthy MZ pairs were significantly smaller than in the discordant pairs.

d Information for the *LSR* gene was extracted from http://www.genecards.org/cgi-bin/carddisp.pl?gene=LSR.

**Table 3:** Results of pathway analysis

*Gene set*	P*-value*	*FDR*	*Nodes*
*Concordant*			
Wnt signaling pathway	0	<5.000e−04	PCDH family, PRKCG, CDH15
Cadherin signaling pathway	0	<5.000e−04	PCDH family, CDH15
Integral to membrane	0	<1.000e−03	HLA-DQB2, SLC6A3, HLA-DMB, PCDH family, CDH15
Integral to plasma membrane	0	0.0005	PCDH family, SLC6A3
Plasma membrane	0.0007	0.03967	PCDH family, IRS1, CDH15
Homophilic cell adhesion	0	<1.000e−03	PCDH family, CDH15
Nervous system development	0	<5.000e−04	PCDH family, ZNF423
Cell adhesion	0	<3.333e−04	PCDH family, CDH15
Calcium ion binding	0	<1.000e−03	PCDH family, CDH15

*Discordant*			
Wnt signaling pathway	0	<1.000e−03	APC2, PCDH family, PPP2R5A, GNB1
Cadherin signaling pathway	0	<5.000e−04	PCDH family
Rapid glucocorticoid signaling	0.0001	0.01167	MAPK11, GNB1
Dopaminergic synapse	0.0002	0.0115	PPP2R5A, MAPK11, GNB1, CACNA1C
IL-2-mediated signaling events	0.0002	0.009833	IRS2, MAPK11, LCK
Thromboxane A2 receptor signaling	0.0002	0.009833	MAPK11, GNB1, LCK
Beta1 adrenergic receptor signaling pathway	0.0017	0.04944	GNB1, CACNA1C
Homophilic cell adhesion	0	<1.000e−03	PCDH family
Nervous system development	0.0001	0.0475	PCDH family

*Healthy*			
HIF-1-alpha transcription factor network (N)	0.0067	0.019	TFRC, ETS1

Abbreviations: FDR, false discovery rate; IL-2, interleukin-2; PCDH, protocadherin.

Gene lists from loci of CpG sites with large intrapair differences in each set of twins (concordant, discordant and healthy) were enriched for the processes shown. For the underlying gene networks, see Figure 1. The full gene lists uploaded for pathway analysis can be found in Supplementary Table 3.
