# Supplementary material for: Genome-wide methylation study on depression: differential methylation and variable methylation in monozygotic twins
Source: Transl Psychiatry. 2015 Apr 28;5(4):e557–. doi: 10.1038/tp.2015.49 (PMC4462612; doi:10.1038/tp.2015.49)
Supplement: Supplementary Information [file tp201549x1.doc]

***Supplementary material***

The next material refers to the manuscript:

***Genome-wide Methylation Study on Depression:***

***Differential Variability and Differential Methylation in Monozygotic Twins***

Authored by:

*Aldo Córdova-Palomera, Mar Fatjó-Vilas, Cristóbal Gastó, Marie-Odile Krebs, Lourdes Fañanás*

**Supplementary Table 1.** 2

**Supplementary Table 2.** 3

**Supplementary Table 3.** 4

**Supplementary Table 4.** 15

**SUPPLEMENTARY TABLE 1.** Comparison of DNA methylation differences in the top-ten DMPs found in discordant MZ twins, with the respective probes in concordant and healthy pairs.

**Notes:** ^a^, superindices next to each probe name indicate whether or not there were (absolute) methylation differences in concordant and healthy pairs; ^1^, absolute intrapair differences in the 4 diagnostic-concordant MZ pairs were significantly smaller than in the discordant pairs; ^2^, absolute intrapair differences in the 7 healthy MZ pairs were significantly smaller than in the discordant pairs; ^b^, statistical comparison of differences in means were performed via the non-parametric Wilcoxon-Mann-Whitney test, hypothesizing that discordant pairs exhibit larger intrapair differences than concordant and healthy pairs; *, statistically significant at p ≤ 0.05; **, statistically significant at p ≤ 0.01.

|  |  | **Mean absolute differences** | | | **Statistical comparisons^b^** | |
| --- | --- | --- | --- | --- | --- | --- |
| **Probe name**  **(TargetID)^a^** | **Gene name**  **(UCSC)** | **Discordant** | **Concordant** | **Healthy** | **Discordant**  **vs**  **Concordant** | **Discordant**  **vs**  **Healthy** |
| **cg06493080^1,2^** | *HOXB7* | 0.08468835 | 0.01770617 | 0.02412389 | 0.004762** | 0.004079** |
| **cg00567749^1^** | *VCAN* | 0.10525752 | 0.03394212 | 0.0730988 | 0.004762** | 0.1171 |
| **cg18974921^1,2^** |  | 0.076364 | 0.01154423 | 0.02788817 | 0.004762** | 0.0005828** |
| **cg14747903^1,2^** | *LSR* | 0.07234575 | 0.02143193 | 0.02212788 | 0.0119* | 0.002165** |
| **cg15696634** |  | 0.07176115 | 0.0549062 | 0.04985944 | 0.2381 | 0.1171 |
| **cg11433980^1^** | *CBR3* | 0.07522222 | 0.0297044 | 0.04339563 | 0.009524** | 0.06876 |
| **cg01122889^1,2^** | *WDR26* | 0.07078792 | 0.03550288 | 0.01986026 | 0.01905* | 0.001166** |
| **cg10550693^1^** | *SYVN1* | 0.07322685 | 0.01658105 | 0.05365276 | 0.01905* | 0.2226 |
| **cg23004466^1^** | *HBP1* | 0.07053208 | 0.0291041 | 0.05270141 | 0.01905* | 0.1171 |
| **cg17798944^1^** | *SNORD43,*  *RPL3* | 0.07204178 | 0.02109322 | 0.05246844 | 0.01905* | 0.183 |

**SUPPLEMENTARY TABLE 2.** Comparison of genome-wide DNA methylation intrapair correlations (Kendall’s tau) across groups of MZ twin pairs.

Values shown correspond to the Kendall’s tau, which was estimated for each twin pair using genome-wide data. **Note:** *, group comparisons were performed using the Wilcoxon-Mann-Whitney test. **Abbreviations:** MZ, monozygotic; SD, standard deviation.

|  | **MZ twin group** | | | **Group comparisons*** | | |
| --- | --- | --- | --- | --- | --- | --- |
|  | **Concordant** | **Discordant** | **Healthy** | **Concordant**  **vs**  **Discordant** | **Concordant**  **vs**  **Healthy** | **Discordant**  **vs**  **Healthy** |
| **Mean (SD)** | 0.9154 (0.0099) | 0.8979 (0.028) | 0.9125 (0.0132) | W = 8,  *p* = 0.4762 | W = 13,  *p* = 0.9273 | W = 26,  *p* = 0.5338 |
| **Range** | 0.9025-0.9264 | 0.8515-0.92 | 0.8902-0.9292 |  |  |  |

**SUPPLEMENTARY TABLE 3.** Variably methylated probes (VMPs) in each diagnostic group of co-twins. As mentioned in the manuscript the VMP threshold selected was a median absolute intrapair difference ≥10% of the methylation fraction. Probes highlighted with yellow colour were located at genes codifying for proteins within the enriched pathways (Figure 1).

**Abbreviations:** TargetID, unique CpG locus identifier from the Illumina CG database; Chr, chromosome; MAPINFO, coordinates (hg19); Median Abs. Diff., median value of the absolute intrapair difference in DNA methylation fraction for the corresponding subset of MZ pairs.

| **Concordant MZ pairs** | | | | | **Discordant MZ pairs** | | | | | **Healthy MZ pairs** | | | | |
| --- | --- | --- | --- | --- | --- | --- | --- | --- | --- | --- | --- | --- | --- | --- |
| ***TargetID*** | ***Chr*** | ***MAPINFO*** | ***Gene name***  ***(UCSC)*** | ***Median***  ***Abs.***  ***Diff.*** | ***TargetID*** | ***Chr*** | ***MAPINFO*** | ***Gene name***  ***(UCSC)*** | ***Median***  ***Abs.***  ***Diff.*** | ***TargetID*** | ***Chr*** | ***MAPINFO*** | ***Gene name***  ***(UCSC)*** | ***Median***  ***Abs.***  ***Diff.*** |
| **cg14341177** | 9 | 95475787 | BICD2;  BICD2 | 0.45 | **cg12053442** | 10 | 124953690 |  | 0.23 | **cg07833104** | 1 | 109797333 | CELSR2 | 0.38 |
| **cg18741962** | 5 | 64920682 | C5orf44;  C5orf44;  C5orf44;  C5orf44;  C5orf44;  TRIM23;  C5orf44;  TRIM23;  C5orf44;  TRIM23 | 0.37 | **cg27453745** | 16 | 86546938 | FOXF1 | 0.18 | **cg24702040** | 1 | 233501790 | KIAA1804 | 0.28 |
| **cg16578226** | 11 | 126055303 |  | 0.24 | **cg02467858** | 15 | 99790901 | TTC23;  TTC23;  TTC23;  TTC23;  TTC23;  LRRC28;  TTC23;  TTC23 | 0.17 | **cg01419577** | 1 | 149170327 |  | 0.19 |
| **cg25987564** | 4 | 6010075 |  | 0.21 | **cg27069263** | 2 | 55278118 | RTN4;  RTN4;  RTN4 | 0.16 | **cg16226866** | 6 | 110418140 |  | 0.16 |
| **cg03548123** | 19 | 1672090 |  | 0.17 | **cg22328771** | 22 | 50709822 | MAPK11 | 0.15 | **cg21164095** | 1 | 32228821 | BAI2 | 0.15 |
| **cg14042190** | 2 | 2547089 |  | 0.17 | **cg17265994** | 11 | 2905024 | CDKN1C;  CDKN1C;  CDKN1C | 0.15 | **cg04737991** | 12 | 105050768 | CHST11 | 0.15 |
| **cg10011799** | 9 | 123554649 | FBXW2;  LOC402377 | 0.17 | **cg22505205** | 6 | 7726983 | BMP6 | 0.15 | **cg18926797** | 14 | 69523229 | DCAF5 | 0.14 |
| **cg23549902** | 7 | 5184155 |  | 0.16 | **cg04870110** | 6 | 27596156 |  | 0.15 | **cg07376282** | 7 | 124404324 | GPR37 | 0.14 |
| **cg19235801** | 10 | 65276945 |  | 0.16 | **cg24348114** | 19 | 35758855 | USF2;  LSR;  LSR;  USF2;  LSR | 0.15 | **cg16361890** | 5 | 179220545 | LTC4S | 0.14 |
| **cg05482498** | 6 | 28866178 |  | 0.15 | **cg00749520** | 19 | 55895972 | RPL28;  RPL28;  RPL28;  RPL28;  RPL28 | 0.15 | **cg02030275** | 20 | 31169126 |  | 0.13 |
| **cg00538591** | 16 | 89008994 | CBFA2T3;  CBFA2T3 | 0.15 | **cg19340909** | 1 | 1821422 | GNB1 | 0.14 | **cg27361134** | 20 | 43439545 | RIMS4 | 0.13 |
| **cg06874323** | 10 | 102986525 |  | 0.15 | **cg00820958** | 8 | 146032149 | ZNF517 | 0.14 | **cg19535096** | 4 | 68751711 |  | 0.13 |
| **cg24820672** | 13 | 112984728 |  | 0.15 | **cg02891664** | 4 | 1004678 | FGFRL1;  FGFRL1 | 0.14 | **cg24056365** | 1 | 110834615 |  | 0.13 |
| **cg14500486** | 6 | 159655392 | FNDC1 | 0.15 | **cg27635110** | 2 | 1926436 | MYT1L | 0.14 | **cg19005485** | 3 | 197272328 | BDH1;  BDH1;  BDH1 | 0.13 |
| **cg26478297** | 19 | 54387435 | PRKCG | 0.14 | **cg05393828** | 1 | 23034799 |  | 0.14 | **cg03271827** | 4 | 726053 | PCGF3 | 0.12 |
| **cg07381806** | 19 | 2094327 | MOBKL2A | 0.14 | **cg03062166** | 9 | 34458659 | DNAI1;  C9orf25 | 0.14 | **cg00014203** | 17 | 7757969 | KDM6B;  TMEM88 | 0.12 |
| **cg02503808** | 4 | 7069936 | GRPEL1 | 0.14 | **cg21141329** | 17 | 48290459 |  | 0.14 | **cg25739938** | 2 | 9610621 | CPSF3 | 0.12 |
| **cg24769969** | 13 | 36051666 | MIR548F5;  NBEA;  MAB21L1 | 0.14 | **cg20482390** | 20 | 62183924 | C20orf195 | 0.14 | **cg04430911** | 1 | 36914349 | OSCP1;  OSCP1 | 0.12 |
| **cg08409113** | 17 | 40937365 | WNK4 | 0.14 | **cg07572579** | 15 | 68116058 |  | 0.14 | **cg15986587** | 19 | 56130797 |  | 0.12 |
| **cg24041453** | 18 | 47792708 | CCDC11 | 0.14 | **cg19430537** | 17 | 74118361 |  | 0.14 | **cg04920917** | 17 | 59539484 | TBX4 | 0.12 |
| **cg19206486** | 19 | 49523046 |  | 0.14 | **cg24356700** | 7 | 100482725 | SRRT;  SRRT;  SRRT;  SRRT | 0.14 | **cg02334333** | 2 | 60687392 | BCL11A;  BCL11A;  BCL11A | 0.12 |
| **cg24082680** | 1 | 63249199 | ATG4C;  ATG4C | 0.14 | **cg05376756** | 1 | 101493828 |  | 0.14 | **cg06361531** | 16 | 68057779 | DUS2L;  DDX28 | 0.12 |
| **cg00325531** | 1 | 75591353 |  | 0.14 | **cg24341498** | 9 | 137217390 | RXRA | 0.14 | **cg23745290** | 4 | 106392021 | PPA2;  PPA2;  PPA2;  PPA2;  PPA2 | 0.12 |
| **cg09649545** | 16 | 85155383 |  | 0.14 | **ch.3.3021133F** | 3 | 153087412 |  | 0.14 | **cg06295495** | 10 | 129948460 |  | 0.12 |
| **cg15795305** | 10 | 102381344 |  | 0.14 | **cg25418748** | 5 | 178977236 | RUFY1 | 0.14 | **cg14927083** | 9 | 132175840 |  | 0.12 |
| **cg05837990** | 7 | 105596483 |  | 0.14 | **ch.6.2893423F** | 6 | 150056792 | NUP43 | 0.13 | **cg12754495** | 3 | 151102703 | P2RY12;  MED12L | 0.12 |
| **cg04661382** | 11 | 109294584 | C11orf87 | 0.14 | **cg05346855** | 1 | 212460169 | PPP2R5A | 0.13 | **cg09183124** | 1 | 173092323 |  | 0.12 |
| **cg19623624** | 10 | 135278901 | LOC619207 | 0.14 | **cg15798563** | 19 | 18436975 |  | 0.13 | **cg15768138** | 2 | 219030752 | CXCR1 | 0.12 |
| **cg01566965** | 4 | 174447847 | HAND2 | 0.14 | **cg14502625** | 5 | 33162283 |  | 0.13 | **cg08202402** | 2 | 240132258 | HDAC4 | 0.12 |
| **cg19664229** | 5 | 1405070 | SLC6A3 | 0.13 | **cg07040834** | 3 | 44063541 |  | 0.13 | **cg15151364** | 1 | 167298762 | POU2F1 | 0.11 |
| **cg22152229** | 5 | 140208954 | PCDHA6;  PCDHA2;  PCDHA1;  PCDHA1;  PCDHA6;  PCDHA5;  PCDHA3;  PCDHA4;  PCDHA6 | 0.13 | **cg07401258** | 21 | 46123590 | C21orf29 | 0.13 | **cg19098932** | 1 | 2345152 | PEX10;  PEX10 | 0.11 |
| **cg10864200** | 4 | 720809 | PCGF3 | 0.13 | **cg12788108** | 17 | 58499300 | C17orf64 | 0.13 | **cg19102955** | 20 | 5928064 | TRMT6 | 0.11 |
| **cg07504200** | 19 | 2889208 |  | 0.13 | **cg02815282** | 11 | 67141135 | LOC100130987;  CLCF1;  CLCF1;  CLCF1 | 0.13 | **cg22278087** | 10 | 21462145 | NEBL | 0.11 |
| **cg07564767** | 4 | 4389668 | D4S234E;  D4S234E | 0.13 | **cg17066594** | 11 | 67764654 | UNC93B1 | 0.13 | **cg02033582** | 17 | 29394814 |  | 0.11 |
| **cg02641337** | 4 | 4867387 |  | 0.13 | **cg01394339** | 6 | 73972288 | KHDC1 | 0.13 | **cg08930843** | 10 | 31182298 | ZNF438;  ZNF438;  ZNF438;  ZNF438;  ZNF438;  ZNF438;  ZNF438;  ZNF438 | 0.11 |
| **cg15572235** | 7 | 5183992 |  | 0.13 | **cg03492747** | 16 | 86543808 | FOXF1 | 0.13 | **cg08534016** | 5 | 35771584 | SPEF2 | 0.11 |
| **cg25970575** | 4 | 6010164 |  | 0.13 | **cg01317586** | 15 | 89921236 | LOC254559 | 0.13 | **cg05946920** | 22 | 38005270 | GGA1;  GGA1;  GGA1 | 0.11 |
| **cg18011273** | 4 | 7203326 | SORCS2 | 0.13 | **cg17605604** | 5 | 102612899 | C5orf30 | 0.13 | **cg04781916** | 2 | 48013473 | MSH6 | 0.11 |
| **cg01960885** | 18 | 24765781 | CHST9 | 0.13 | **cg21860429** | 6 | 105389544 |  | 0.13 | **cg14187687** | 14 | 55249770 | SAMD4A;  SAMD4A;  SAMD4A | 0.11 |
| **cg14742937** | 8 | 39172097 | ADAM5P | 0.13 | **cg17059694** | 2 | 105473800 |  | 0.13 | **cg10506318** | 5 | 60239580 | ERCC8;  NDUFAF2 | 0.11 |
| **cg09026360** | 3 | 46742703 | TMIE | 0.13 | **cg19319069** | 7 | 129593010 | UBE2H;  UBE2H | 0.13 | **cg25749565** | 7 | 61971401 |  | 0.11 |
| **cg16702083** | 20 | 62328427 | TNFRSF6B;  TNFRSF6B | 0.13 | **cg01547051** | 7 | 150777654 | FASTK;  FASTK | 0.13 | **cg18784409** | 11 | 67868331 | CHKA;  CHKA | 0.11 |
| **cg08355456** | 11 | 67383691 |  | 0.13 | **cg15744668** | 2 | 175207395 |  | 0.13 | **cg01054652** | 11 | 72246863 |  | 0.11 |
| **cg24690094** | 11 | 67383802 |  | 0.13 | **cg07376381** | 1 | 32741518 | LCK;  LCK | 0.12 | **cg10371414** | 16 | 79451857 |  | 0.11 |
| **cg16896868** | 13 | 110319562 |  | 0.13 | **cg06952129** | 5 | 80362130 | RASGRF2 | 0.12 | **cg24728803** | 14 | 99742268 |  | 0.11 |
| **cg04364540** | 10 | 116582297 | FAM160B1;  FAM160B1 | 0.13 | **cg05005382** | 16 | 841550 | CHTF18 | 0.12 | **cg18309895** | 10 | 105445329 | SH3PXD2A | 0.11 |
| **cg02048922** | 8 | 143546858 | BAI1 | 0.13 | **cg10958362** | 5 | 2754016 | C5orf38 | 0.12 | **cg23007425** | 14 | 105600994 |  | 0.11 |
| **cg00732845** | 7 | 155244727 |  | 0.13 | **cg23760945** | 19 | 11665140 | ELOF1 | 0.12 | **cg26294145** | 14 | 106086764 |  | 0.11 |
| **cg02680710** | 6 | 169310568 |  | 0.13 | **cg17013691** | 5 | 14380323 | TRIO | 0.12 | **cg18279004** | 3 | 111802305 |  | 0.11 |
| **cg01392179** | 5 | 170848123 | FGF18 | 0.13 | **cg26279152** | 13 | 20356101 | PSPC1;  PSPC1 | 0.12 | **cg11842502** | 6 | 111862202 |  | 0.11 |
| **cg06506560** | 17 | 38474741 | RARA;  RARA;  RARA | 0.12 | **cg01710189** | 8 | 22454888 | PDLIM2 | 0.12 | **cg16595484** | 3 | 122512170 | HSPBAP1 | 0.11 |
| **cg07633317** | 16 | 49732623 | ZNF423 | 0.12 | **cg25997110** | 19 | 31842115 |  | 0.12 | **cg12405599** | 3 | 128370463 | RPN1 | 0.11 |
| **cg01413281** | 10 | 101989592 | CHUK | 0.12 | **cg19582538** | 6 | 38127734 |  | 0.12 | **cg25489105** | 3 | 129155354 | MBD4 | 0.11 |
| **cg01558909** | 16 | 215845 | HBM | 0.12 | **cg14675896** | 19 | 38830208 | CATSPERG | 0.12 | **cg25435819** | 1 | 149149385 |  | 0.11 |
| **cg00345425** | 4 | 2070915 |  | 0.12 | **cg25595442** | 1 | 39981352 | BMP8A | 0.12 | **cg01942558** | 2 | 152214027 | TNFAIP6 | 0.11 |
| **cg07296995** | 5 | 2129767 |  | 0.12 | **cg10463299** | 20 | 54987330 | CASS4;  CASS4;  CASS4;  CASS4;  CASS4;  CASS4;  CASS4 | 0.12 | **cg06022561** | 2 | 242702553 | D2HGDH | 0.11 |
| **cg11834635** | 4 | 2402019 | ZFYVE28 | 0.12 | **cg09194930** | 16 | 56651283 | MT1L | 0.12 | **cg25578781** | 11 | 128446070 | ETS1 | 0.1 |
| **cg20932426** | 1 | 2415221 | PLCH2 | 0.12 | **cg19233923** | 11 | 63753598 | OTUB1;  OTUB1;  OTUB1 | 0.12 | **cg09470983** | 3 | 195805948 | TFRC;  TFRC | 0.1 |
| **cg01157718** | 6 | 3255870 |  | 0.12 | **cg00687674** | 15 | 69373260 | MIR548H4;  TMEM84 | 0.12 | **cg09571972** | 6 | 2104322 | GMDS | 0.1 |
| **cg20295248** | 20 | 5485270 | LOC149837 | 0.12 | **cg02143067** | 7 | 73081771 | VPS37D | 0.12 | **cg13196550** | 11 | 2290105 | ASCL2 | 0.1 |
| **cg20663042** | 17 | 6734940 | TEKT1 | 0.12 | **cg23439743** | 16 | 73099892 |  | 0.12 | **cg15964132** | 11 | 3175636 | OSBPL5;  OSBPL5;  OSBPL5 | 0.1 |
| **cg18544450** | 19 | 14264896 | LPHN1;  LPHN1 | 0.12 | **cg01423820** | 14 | 77228690 | VASH1;  VASH1 | 0.12 | **cg18276112** | 7 | 4755032 | FOXK1 | 0.1 |
| **cg20935784** | 13 | 19919283 | LOC100101938 | 0.12 | **cg19473369** | 15 | 99190447 |  | 0.12 | **cg26877678** | 17 | 25856540 | KSR1 | 0.1 |
| **cg14560110** | 17 | 20799470 | CCDC144NL | 0.12 | **cg08194011** | 14 | 101996442 |  | 0.12 | **cg27309564** | 7 | 27223097 | HOXA11 | 0.1 |
| **cg25602603** | 19 | 22320744 |  | 0.12 | **cg05495546** | 5 | 113390551 |  | 0.12 | **cg05619116** | 3 | 27754569 |  | 0.1 |
| **cg15624813** | 10 | 23492866 |  | 0.12 | **cg18825597** | 8 | 144506958 |  | 0.12 | **cg07023317** | 8 | 28961315 | KIF13B | 0.1 |
| **cg00889945** | 22 | 25202552 | SGSM1;  SGSM1;  SGSM1;  SGSM1 | 0.12 | **cg26567012** | 5 | 151202148 | GLRA1;  GLRA1 | 0.12 | **cg02640604** | 16 | 29690271 | QPRT | 0.1 |
| **cg23591853** | 2 | 27712996 | IFT172 | 0.12 | **cg13303229** | 5 | 154136254 | LARP1;  LARP1 | 0.12 | **cg00578614** | 6 | 30070403 |  | 0.1 |
| **cg04546413** | 19 | 29218101 |  | 0.12 | **cg12698713** | 6 | 167836850 |  | 0.12 | **cg03706056** | 21 | 37437565 | SETD4 | 0.1 |
| **cg24448840** | 6 | 30539883 | ABCF1;  ABCF1 | 0.12 | **cg21625568** | 5 | 173738479 |  | 0.12 | **cg00475235** | 17 | 54236118 | ANKFN1 | 0.1 |
| **cg19927508** | 16 | 31439191 | COX6A2 | 0.12 | **cg05777976** | 11 | 2163538 | IGF2AS;  INS-IGF2;  IGF2;  IGF2;  IGF2AS | 0.11 | **cg18830993** | 14 | 56672508 | PELI2 | 0.1 |
| **cg00547425** | 6 | 31829960 | NEU1 | 0.12 | **cg25133685** | 6 | 29013336 | OR2W1 | 0.11 | **cg03447530** | 13 | 60970210 | TDRD3;  TDRD3;  TDRD3 | 0.1 |
| **cg23043245** | 6 | 34434076 | PACSIN1 | 0.12 | **cg26386788** | 7 | 36430296 | ANLN;  KIAA0895 | 0.11 | **cg10408731** | 7 | 65214843 | CCT6P1 | 0.1 |
| **cg25757140** | 19 | 38039469 |  | 0.12 | **cg15141401** | 11 | 55371077 | OR4C11 | 0.11 | **cg09140723** | 10 | 72594465 | SGPL1 | 0.1 |
| **cg23303369** | 20 | 44641161 | MMP9 | 0.12 | **cg09985739** | 13 | 67621714 | PCDH9;  PCDH9 | 0.11 | **cg04286455** | 5 | 77803462 | LHFPL2 | 0.1 |
| **cg07104919** | 11 | 46958073 | C11orf49;  C11orf49;  C11orf49;  C11orf49 | 0.12 | **cg02204262** | 5 | 140201746 | PCDHA2;  PCDHA1;  PCDHA1;  PCDHA3;  PCDHA4;  PCDHA5;  PCDHA5 | 0.11 | **cg15436606** | 4 | 87881747 | AFF1 | 0.1 |
| **cg16378063** | 17 | 47329552 |  | 0.12 | **cg11349969** | 2 | 169028056 | STK39 | 0.11 | **cg03170665** | 2 | 114333496 | FAM138B | 0.1 |
| **cg17328716** | 7 | 56516243 | LOC650226 | 0.12 | **cg20706311** | 10 | 724510 | DIP2C | 0.11 | **cg05631230** | 11 | 116522252 |  | 0.1 |
| **cg18313094** | 19 | 57633694 | USP29 | 0.12 | **cg02663945** | 1 | 861629 | SAMD11 | 0.11 | **cg03579446** | 8 | 126216633 | NSMCE2 | 0.1 |
| **cg01781617** | 17 | 60216199 |  | 0.12 | **cg22510943** | 19 | 2456368 | LMNB2 | 0.11 | **cg22825487** | 6 | 133055183 | VNN3;  VNN3 | 0.1 |
| **cg00833479** | 7 | 62528852 |  | 0.12 | **cg21195290** | 9 | 2621094 | VLDLR;  VLDLR;  FLJ35024 | 0.11 | **cg06255004** | 9 | 139580799 | AGPAT2;  AGPAT2 | 0.1 |
| **cg22683036** | 1 | 63249209 | ATG4C;  ATG4C | 0.12 | **cg16117781** | 1 | 7991159 | TNFRSF9 | 0.11 | **cg09727050** | 2 | 152214177 | TNFAIP6;  TNFAIP6 | 0.1 |
| **cg27457290** | 2 | 64246845 | VPS54;  VPS54 | 0.12 | **cg21653586** | 11 | 10530636 |  | 0.11 | **cg14249174** | 4 | 153814351 | ARFIP1;  ARFIP1;  ARFIP1 | 0.1 |
| **cg23188684** | 11 | 67383651 |  | 0.12 | **cg20760116** | 3 | 13324924 |  | 0.11 | **cg14257907** | 1 | 165853483 | UCK2 | 0.1 |
| **cg01066472** | 1 | 75591029 |  | 0.12 | **cg10861135** | 9 | 14345348 |  | 0.11 | **cg00181432** | 3 | 194393818 | LSG1 | 0.1 |
| **cg10937408** | 17 | 77478569 | HRNBP3 | 0.12 | **ch.3.382096F** | 3 | 16768672 |  | 0.11 |  |  |  |  |  |
| **cg05696877** | 1 | 79088769 | IFI44L | 0.12 | **cg02130266** | 22 | 17078353 |  | 0.11 |  |  |  |  |  |
| **cg05754819** | 10 | 79397944 | KCNMA1;  KCNMA1;  KCNMA1;  KCNMA1 | 0.12 | **cg01727302** | 7 | 24321351 |  | 0.11 |  |  |  |  |  |
| **cg19597498** | 3 | 96495651 |  | 0.12 | **cg19857379** | 1 | 25105430 | CLIC4 | 0.11 |  |  |  |  |  |
| **cg13994504** | 10 | 99338056 | ANKRD2;  ANKRD2 | 0.12 | **cg27319123** | 11 | 30038286 | KCNA4;  KCNA4 | 0.11 |  |  |  |  |  |
| **cg02193956** | 4 | 103680201 | MANBA | 0.12 | **cg26558420** | 20 | 30331315 | TPX2 | 0.11 |  |  |  |  |  |
| **cg02909372** | 10 | 104193735 | CUEDC2 | 0.12 | **cg04194272** | 20 | 33683537 |  | 0.11 |  |  |  |  |  |
| **cg20181887** | 12 | 123753272 | CDK2AP1 | 0.12 | **cg12287945** | 19 | 35758300 | LSR;  LSR;  LSR | 0.11 |  |  |  |  |  |
| **cg14035787** | 8 | 128428308 | POU5F1B | 0.12 | **cg08343347** | 4 | 36076000 | ARAP2 | 0.11 |  |  |  |  |  |
| **cg11446507** | 10 | 132692284 |  | 0.12 | **cg05644223** | 21 | 36412119 | C21orf96;  RUNX1 | 0.11 |  |  |  |  |  |
| **cg14316629** | 6 | 168436353 | KIF25;KIF25 | 0.12 | **cg03593057** | 17 | 40128637 | DNAJC7;  DNAJC7;  CNP;  DNAJC7 | 0.11 |  |  |  |  |  |
| **cg11953245** | 1 | 228291591 | C1orf35 | 0.12 | **cg22131547** | 17 | 44274436 |  | 0.11 |  |  |  |  |  |
| **cg03638120** | 6 | 32729720 | HLA-DQB2 | 0.11 | **cg04609859** | 17 | 46655736 | HOXB4;  HOXB4 | 0.11 |  |  |  |  |  |
| **cg09017117** | 6 | 32904419 | HLA-DMB | 0.11 | **cg03413884** | 7 | 54610928 | VSTM2A | 0.11 |  |  |  |  |  |
| **cg00610021** | 22 | 45809244 | SMC1B;  RIBC2 | 0.11 | **cg10648815** | 19 | 55013549 | LAIR2;  LAIR2 | 0.11 |  |  |  |  |  |
| **cg25103043** | 19 | 54385333 | PRKCG | 0.11 | **cg20724428** | 3 | 57997675 | FLNB;  FLNB;  FLNB;  FLNB | 0.11 |  |  |  |  |  |
| **cg00653460** | 3 | 101406220 | RPL24 | 0.11 | **cg01056135** | 18 | 61253151 | SERPINB13 | 0.11 |  |  |  |  |  |
| **cg01512854** | 5 | 140480967 | PCDHB3 | 0.11 | **cg18803079** | 1 | 64014643 | EFCAB7;  DLEU2L | 0.11 |  |  |  |  |  |
| **cg03828328** | 5 | 140744556 | PCDHGA4;  PCDHGA2;  PCDHGA5;  PCDHGB2;  PCDHGA1;  PCDHGB1;  PCDHGA3;  PCDHGA5 | 0.11 | **cg17983571** | 10 | 65186953 | JMJD1C | 0.11 |  |  |  |  |  |
| **cg00758915** | 5 | 140773539 | PCDHGA4;  PCDHGA6;  PCDHGA1;  PCDHGA5;  PCDHGB1;  PCDHGB4;  PCDHGA3;  PCDHGA2;  PCDHGA8;  PCDHGA7;  PCDHGB2;  PCDHGA8;  PCDHGB3 | 0.11 | **cg08960352** | 12 | 68043561 | DYRK2;  DYRK2 | 0.11 |  |  |  |  |  |
| **cg07613945** | 5 | 140787430 | PCDHGA4;  PCDHGA6;  PCDHGA9;  PCDHGA1;  PCDHGA8;  PCDHGA5;  PCDHGB1;  PCDHGB6;  PCDHGB4;  PCDHGA3;  PCDHGA2;  PCDHGA7;  PCDHGB2;  PCDHGB6;  PCDHGB5;  PCDHGB3 | 0.11 | **cg10934870** | 2 | 70488000 | PCYOX1 | 0.11 |  |  |  |  |  |
| **cg05413628** | 16 | 1521656 | CLCN7;  CLCN7 | 0.11 | **cg11806367** | 5 | 71146767 |  | 0.11 |  |  |  |  |  |
| **cg05617980** | 4 | 3373405 | RGS12;  RGS12;  RGS12 | 0.11 | **cg09013626** | 3 | 72495672 | RYBP | 0.11 |  |  |  |  |  |
| **cg27436118** | 16 | 4729905 | MGRN1;  MGRN1;  MGRN1;  MGRN1 | 0.11 | **cg07786668** | 16 | 73092391 | ZFHX3 | 0.11 |  |  |  |  |  |
| **cg10831901** | 20 | 5485294 | LOC149837 | 0.11 | **cg18007641** | 4 | 74641828 |  | 0.11 |  |  |  |  |  |
| **cg24834921** | 1 | 5779237 |  | 0.11 | **cg02320862** | 17 | 75370284 | SEPT9;  SEPT9;  SEPT9;  SEPT9 | 0.11 |  |  |  |  |  |
| **cg17360233** | 16 | 10479841 |  | 0.11 | **cg17411546** | 10 | 75410026 | SYNPO2L;  SYNPO2L | 0.11 |  |  |  |  |  |
| **cg23436576** | 8 | 11204132 | TDH | 0.11 | **cg20273576** | 17 | 79108139 | AATK;  MIR1250 | 0.11 |  |  |  |  |  |
| **cg00099295** | 3 | 12597720 | MKRN2 | 0.11 | **cg00021600** | 16 | 84003607 | NECAB2 | 0.11 |  |  |  |  |  |
| **cg07582862** | 3 | 13549288 |  | 0.11 | **cg15575098** | 1 | 84304612 |  | 0.11 |  |  |  |  |  |
| **cg16503797** | 18 | 19476805 |  | 0.11 | **cg00350932** | 2 | 86335912 | PTCD3 | 0.11 |  |  |  |  |  |
| **cg20430047** | 20 | 20257861 | C20orf26 | 0.11 | **cg08152839** | 15 | 92936232 | ST8SIA2 | 0.11 |  |  |  |  |  |
| **cg08872493** | 1 | 23521417 | HTR1D | 0.11 | **cg00014638** | 10 | 94113651 |  | 0.11 |  |  |  |  |  |
| **cg01363170** | 7 | 27236674 | HOXA13 | 0.11 | **cg00730266** | 7 | 94537716 | PPP1R9A;  PPP1R9A;  PPP1R9A;  PPP1R9A;  PPP1R9A | 0.11 |  |  |  |  |  |
| **cg22572476** | 6 | 28601324 |  | 0.11 | **cg14583550** | 9 | 98071630 | FANCC | 0.11 |  |  |  |  |  |
| **cg10582608** | 6 | 28664190 |  | 0.11 | **cg02657244** | 4 | 99183198 | RAP1GDS1;  RAP1GDS1;  RAP1GDS1;  RAP1GDS1;  RAP1GDS1;  RAP1GDS1 | 0.11 |  |  |  |  |  |
| **cg03364486** | 8 | 29172968 |  | 0.11 | **cg07176677** | 10 | 101771161 | DNMBP | 0.11 |  |  |  |  |  |
| **cg17977304** | 6 | 29623992 | MOG;  MOG;  MOG;  MOG;  MOG;  MOG;  MOG;  MOG;  MOG;  MOG;  MOG | 0.11 | **cg11890239** | 8 | 103541014 |  | 0.11 |  |  |  |  |  |
| **cg01444849** | 16 | 34430837 |  | 0.11 | **cg22562498** | 5 | 111095202 | C5orf13;  C5orf13;  C5orf13;  C5orf13 | 0.11 |  |  |  |  |  |
| **cg15754660** | 7 | 34699393 | NPSR1;  NPSR1 | 0.11 | **cg13292470** | 13 | 112718952 |  | 0.11 |  |  |  |  |  |
| **cg22252041** | 6 | 34724756 | SNRPC;  SNRPC | 0.11 | **cg09656541** | 10 | 115932823 | C10orf118 | 0.11 |  |  |  |  |  |
| **cg24661236** | 6 | 36930062 | PI16 | 0.11 | **cg16301890** | 10 | 119301464 | EMX2;  EMX2OS;  EMX2 | 0.11 |  |  |  |  |  |
| **cg14707231** | 5 | 43001138 |  | 0.11 | **cg05020510** | 8 | 120428057 | NOV | 0.11 |  |  |  |  |  |
| **cg24236938** | 6 | 43424191 | DLK2;  DLK2 | 0.11 | **cg00014112** | 2 | 127413530 | GYPC;  GYPC | 0.11 |  |  |  |  |  |
| **cg27665146** | 15 | 51369926 | TNFAIP8L3 | 0.11 | **cg14634918** | 7 | 127449997 | SND1 | 0.11 |  |  |  |  |  |
| **cg15078822** | 7 | 52341469 |  | 0.11 | **cg19028099** | 8 | 141273325 | TRAPPC9;  TRAPPC9 | 0.11 |  |  |  |  |  |
| **cg05655885** | 7 | 56515601 | LOC650226 | 0.11 | **cg07387429** | 1 | 153234809 |  | 0.11 |  |  |  |  |  |
| **cg05516272** | 11 | 66311334 | ZDHHC24 | 0.11 | **cg12340343** | 7 | 155897383 |  | 0.11 |  |  |  |  |  |
| **cg24086348** | 17 | 67138138 | ABCA6 | 0.11 | **cg19070856** | 5 | 158534530 |  | 0.11 |  |  |  |  |  |
| **cg21272279** | 12 | 75785232 | GLIPR1L2 | 0.11 | **cg06353725** | 1 | 162036019 |  | 0.11 |  |  |  |  |  |
| **cg22252999** | 4 | 76996414 | ART3;  ART3;  ART3 | 0.11 | **cg11256764** | 5 | 180085446 |  | 0.11 |  |  |  |  |  |
| **cg13560853** | 12 | 78710064 |  | 0.11 | **cg06346838** | 19 | 1466162 | APC2 | 0.1 |  |  |  |  |  |
| **cg17256697** | 7 | 81240257 |  | 0.11 | **cg10031793** | 12 | 2734257 | CACNA1C;  CACNA1C;  CACNA1C;  CACNA1C;  CACNA1C;  CACNA1C;  CACNA1C;  CACNA1C;  CACNA1C;  CACNA1C;  CACNA1C;  CACNA1C;  CACNA1C;  CACNA1C;  CACNA1C;  CACNA1C;  CACNA1C;  CACNA1C;  CACNA1C;  CACNA1C;  CACNA1C;  CACNA1C;  CACNA1C | 0.1 |  |  |  |  |  |
| **cg03566513** | 12 | 81332744 | LIN7A | 0.11 | **cg14555127** | 7 | 35841578 | SEPT7;  SEPT7 | 0.1 |  |  |  |  |  |
| **cg06102678** | 8 | 81491328 |  | 0.11 | **cg01577933** | 1 | 37498025 | GRIK3 | 0.1 |  |  |  |  |  |
| **cg02687418** | 11 | 85393765 |  | 0.11 | **cg11153485** | 13 | 110431612 | IRS2 | 0.1 |  |  |  |  |  |
| **cg01142721** | 10 | 85985448 | LRIT2 | 0.11 | **cg22083790** | 12 | 133532978 | ZNF605;  ZNF605 | 0.1 |  |  |  |  |  |
| **cg27640988** | 2 | 86850406 | RNF103;  RNF103 | 0.11 | **cg02471078** | 11 | 688159 | DEAF1 | 0.1 |  |  |  |  |  |
| **cg06471402** | 16 | 89259957 | CDH15 | 0.11 | **cg22735391** | 8 | 1238536 |  | 0.1 |  |  |  |  |  |
| **cg17915676** | 2 | 98928898 | VWA3B | 0.11 | **cg01072217** | 7 | 1275483 | UNCX | 0.1 |  |  |  |  |  |
| **cg20126546** | 12 | 101188278 | ANO4 | 0.11 | **cg16327326** | 16 | 1662895 | IFT140 | 0.1 |  |  |  |  |  |
| **cg01477971** | 2 | 103405951 | TMEM182 | 0.11 | **cg08634464** | 19 | 2901147 | ZNF57 | 0.1 |  |  |  |  |  |
| **cg18975804** | 14 | 107210810 |  | 0.11 | **cg11037134** | 19 | 3185679 | NCLN | 0.1 |  |  |  |  |  |
| **cg06059616** | 8 | 109092720 | RSPO2 | 0.11 | **cg10013169** | 6 | 5997028 |  | 0.1 |  |  |  |  |  |
| **cg16288318** | 13 | 112986927 |  | 0.11 | **cg14755414** | 2 | 9531144 | ASAP2;  ASAP2 | 0.1 |  |  |  |  |  |
| **cg00698771** | 1 | 113285940 |  | 0.11 | **cg22187251** | 6 | 10529935 | GCNT2 | 0.1 |  |  |  |  |  |
| **cg10665891** | 12 | 117042917 |  | 0.11 | **cg11949262** | 7 | 20816184 |  | 0.1 |  |  |  |  |  |
| **cg12029105** | 12 | 120032623 | TMEM233 | 0.11 | **cg21484863** | 6 | 21131734 | CDKAL1 | 0.1 |  |  |  |  |  |
| **cg01416111** | 7 | 120590947 | ING3;  ING3;  ING3;  ING3 | 0.11 | **cg07108927** | 18 | 24283669 | LOC728606 | 0.1 |  |  |  |  |  |
| **cg06379361** | 12 | 120755460 |  | 0.11 | **cg12114985** | 19 | 49127660 | SPHK2 | 0.1 |  |  |  |  |  |
| **cg25196975** | 11 | 124738119 | ROBO3 | 0.11 | **cg06770993** | 3 | 49158462 | USP19 | 0.1 |  |  |  |  |  |
| **cg13982956** | 9 | 128521976 | PBX3;  PBX3;  PBX3;  PBX3 | 0.11 | **cg18000800** | 11 | 63390969 |  | 0.1 |  |  |  |  |  |
| **cg16609957** | 5 | 132161867 | SHROOM1;  SHROOM1 | 0.11 | **cg00865774** | 12 | 66219177 | RPSAP52;  HMGA2;  HMGA2 | 0.1 |  |  |  |  |  |
| **cg24413339** | 10 | 135237754 | SPRN | 0.11 | **cg02666610** | 11 | 67499431 |  | 0.1 |  |  |  |  |  |
| **cg10184328** | 7 | 138349158 | SVOPL;  SVOPL | 0.11 | **cg05157098** | 12 | 72059293 | THAP2 | 0.1 |  |  |  |  |  |
| **cg06339924** | 3 | 140395771 | TRIM42 | 0.11 | **cg09169335** | 15 | 73659861 | HCN4 | 0.1 |  |  |  |  |  |
| **cg22692549** | 1 | 149145243 |  | 0.11 | **cg14480262** | 14 | 73703844 | PAPLN | 0.1 |  |  |  |  |  |
| **cg16609021** | 7 | 157754648 | PTPRN2;  PTPRN2;  PTPRN2 | 0.11 | **cg01697989** | 15 | 79574209 | ANKRD34C | 0.1 |  |  |  |  |  |
| **cg01860370** | 7 | 158785768 |  | 0.11 | **cg11963436** | 10 | 93567261 | TNKS2 | 0.1 |  |  |  |  |  |
| **cg07042832** | 3 | 170136539 | CLDN11 | 0.11 | **cg20500854** | 14 | 99707650 | BCL11B;  BCL11B | 0.1 |  |  |  |  |  |
| **cg14653136** | 1 | 197877786 |  | 0.11 | **cg06579481** | 7 | 104621597 |  | 0.1 |  |  |  |  |  |
| **cg17858100** | 1 | 207224510 | YOD1 | 0.11 | **cg24576298** | 7 | 108137995 | PNPLA8 | 0.1 |  |  |  |  |  |
| **cg07308161** | 2 | 211090487 | ACADL | 0.11 | **cg22031999** | 2 | 109407014 | CCDC138 | 0.1 |  |  |  |  |  |
| **cg17379932** | 10 | 12085598 | UPF2;  UPF2 | 0.1 | **cg25039830** | 8 | 117785039 | UTP23 | 0.1 |  |  |  |  |  |
| **cg16208053** | 10 | 135379215 | SYCE1;  SYCE1;  SYCE1 | 0.1 | **cg10957918** | 12 | 125473823 | DHX37 | 0.1 |  |  |  |  |  |
| **cg19928377** | 5 | 140249735 | PCDHA6;  PCDHA2;  PCDHA1;  PCDHA9;  PCDHA7;  PCDHA1;  PCDHA6;  PCDHA5;  PCDHA11;  PCDHA11;  PCDHA10;  PCDHA3;  PCDHA4;  PCDHA10;  PCDHA8 | 0.1 | **cg04760493** | 4 | 128981486 | LARP1B;  LARP1B;  LARP1B | 0.1 |  |  |  |  |  |
| **cg03298167** | 2 | 227663911 | IRS1 | 0.1 | **cg11716073** | 8 | 142182166 | DENND3 | 0.1 |  |  |  |  |  |
| **cg05849772** | 16 | 303182 | ITFG3 | 0.1 | **cg04405926** | 1 | 150208726 | ANP32E;  ANP32E | 0.1 |  |  |  |  |  |
| **cg14825735** | 6 | 1594066 |  | 0.1 | **cg17479576** | 4 | 152424074 | FAM160A1 | 0.1 |  |  |  |  |  |
| **cg02937671** | 4 | 2366485 | ZFYVE28 | 0.1 | **cg20620272** | 3 | 167968129 | C3orf50 | 0.1 |  |  |  |  |  |
| **cg01168584** | 18 | 2903245 | EMILIN2 | 0.1 |  |  |  |  |  |  |  |  |  |  |
| **cg02052762** | 17 | 4090525 | ANKFY1 | 0.1 |  |  |  |  |  |  |  |  |  |  |
| **cg05274323** | 4 | 4386745 | D4S234E;  D4S234E | 0.1 |  |  |  |  |  |  |  |  |  |  |
| **cg16763089** | 20 | 5485284 | LOC149837 | 0.1 |  |  |  |  |  |  |  |  |  |  |
| **cg02744880** | 19 | 11592200 | ELAVL3;  ELAVL3 | 0.1 |  |  |  |  |  |  |  |  |  |  |
| **cg04388792** | 19 | 12707977 | ZNF490 | 0.1 |  |  |  |  |  |  |  |  |  |  |
| **cg00590063** | 19 | 17918795 | B3GNT3 | 0.1 |  |  |  |  |  |  |  |  |  |  |
| **cg07161397** | 1 | 18431241 |  | 0.1 |  |  |  |  |  |  |  |  |  |  |
| **cg19830270** | 19 | 22193349 | ZNF208 | 0.1 |  |  |  |  |  |  |  |  |  |  |
| **cg13367219** | 12 | 28764033 |  | 0.1 |  |  |  |  |  |  |  |  |  |  |
| **cg09266924** | 15 | 29034669 |  | 0.1 |  |  |  |  |  |  |  |  |  |  |
| **cg10310917** | 6 | 29815787 |  | 0.1 |  |  |  |  |  |  |  |  |  |  |
| **cg10821226** | 6 | 29818277 |  | 0.1 |  |  |  |  |  |  |  |  |  |  |
| **cg08667128** | 17 | 29886885 | MIR193A | 0.1 |  |  |  |  |  |  |  |  |  |  |
| **cg15951466** | 6 | 30079192 | TRIM31 | 0.1 |  |  |  |  |  |  |  |  |  |  |
| **cg06957003** | 8 | 30765641 |  | 0.1 |  |  |  |  |  |  |  |  |  |  |
| **cg20653128** | 17 | 40936820 | WNK4 | 0.1 |  |  |  |  |  |  |  |  |  |  |
| **cg06059360** | 3 | 42657618 | NKTR | 0.1 |  |  |  |  |  |  |  |  |  |  |
| **cg07658590** | 21 | 46963587 | SLC19A1 | 0.1 |  |  |  |  |  |  |  |  |  |  |
| **cg23272978** | 11 | 65315030 | LTBP3;  LTBP3;  LTBP3 | 0.1 |  |  |  |  |  |  |  |  |  |  |
| **cg13578160** | 7 | 72813978 |  | 0.1 |  |  |  |  |  |  |  |  |  |  |
| **cg14989243** | 6 | 76203530 | FILIP1 | 0.1 |  |  |  |  |  |  |  |  |  |  |
| **cg26577017** | 8 | 80523191 | STMN2 | 0.1 |  |  |  |  |  |  |  |  |  |  |
| **cg03077331** | 17 | 80693076 | FN3K | 0.1 |  |  |  |  |  |  |  |  |  |  |
| **cg07951728** | 16 | 86331813 |  | 0.1 |  |  |  |  |  |  |  |  |  |  |
| **cg13004509** | 11 | 93063662 | CCDC67 | 0.1 |  |  |  |  |  |  |  |  |  |  |
| **cg27469738** | 10 | 99338074 | ANKRD2;  ANKRD2 | 0.1 |  |  |  |  |  |  |  |  |  |  |
| **cg06779009** | 14 | 102094299 |  | 0.1 |  |  |  |  |  |  |  |  |  |  |
| **cg24933060** | 7 | 102155527 |  | 0.1 |  |  |  |  |  |  |  |  |  |  |
| **cg14877637** | 8 | 104032876 | ATP6V1C1 | 0.1 |  |  |  |  |  |  |  |  |  |  |
| **cg14646244** | 7 | 107300287 | SLC26A4;  LOC286002 | 0.1 |  |  |  |  |  |  |  |  |  |  |
| **cg00487142** | 3 | 109056357 | DPPA4 | 0.1 |  |  |  |  |  |  |  |  |  |  |
| **cg07180125** | 10 | 114039492 |  | 0.1 |  |  |  |  |  |  |  |  |  |  |
| **cg26928972** | 3 | 122043799 | CSTA | 0.1 |  |  |  |  |  |  |  |  |  |  |
| **cg05034195** | 2 | 129659834 |  | 0.1 |  |  |  |  |  |  |  |  |  |  |
| **cg11942181** | 2 | 129659946 |  | 0.1 |  |  |  |  |  |  |  |  |  |  |
| **cg01803766** | 11 | 131561541 | NTM | 0.1 |  |  |  |  |  |  |  |  |  |  |
| **cg26365014** | 10 | 134622019 |  | 0.1 |  |  |  |  |  |  |  |  |  |  |
| **cg04561804** | 3 | 169683976 | LOC100128164;  SEC62;  LOC100128164 | 0.1 |  |  |  |  |  |  |  |  |  |  |
| **cg21120436** | 3 | 173302303 | NLGN1 | 0.1 |  |  |  |  |  |  |  |  |  |  |
| **cg25984400** | 2 | 177001883 |  | 0.1 |  |  |  |  |  |  |  |  |  |  |
| **cg11969330** | 4 | 187125413 | CYP4V2 | 0.1 |  |  |  |  |  |  |  |  |  |  |
| **cg03523835** | 1 | 207223119 | YOD1 | 0.1 |  |  |  |  |  |  |  |  |  |  |
| **cg11336382** | 1 | 228658646 |  | 0.1 |  |  |  |  |  |  |  |  |  |  |
| **cg22700015** | 1 | 228743131 |  | 0.1 |  |  |  |  |  |  |  |  |  |  |
| **cg00793935** | 2 | 234847769 | TRPM8 | 0.1 |  |  |  |  |  |  |  |  |  |  |

**SUPPLEMENTARY TABLE 4.** Probabilities of having randomly sampled gene name lists from the lists of VMPs for concordant, discordant and healthy MZ pairs.

The column “Number of probes” indicates how many probes in the whole DNA methylation array contain an annotation for the corresponding gene. For each gene name shown, the value in “*p* of random sampling” corresponds to the probability of getting a probe with that gene name if one randomly samples 221, 175 or 85 probes (namely, the number of VMPs for each group). For instance, as shown in the first row of the concordant set, the probability of getting a probe with the annotation *ADAM5P* if one randomly selects 221 out of the > 450,000 probes included in the analyses (chromosomes 1-22 in the Illumina array) is around 3×10^-4^. The FDR-adjusted *p*-value corrects the previous probability estimate to account for the fact that there were several genes in each gene list.

| **CONCORDANT**  **(221 probes)** | | | | **DISCORDANT**  **(175 probes)** | | | | **HEALTHY**  **(85 probes)** | | | |
| --- | --- | --- | --- | --- | --- | --- | --- | --- | --- | --- | --- |
| **Gene name**  **(UCSC)** | **Number**  **of**  **probes** | ***p* of**  **random**  **sampling** | **FDR-**  **adjusted**  ***p*** | **Gene name**  **(UCSC)** | **Number**  **of**  **probes** | ***p* of**  **random**  **sampling** | **FDR-**  **adjusted**  ***p*** | **Gene name**  **(UCSC)** | **Number**  **of**  **probes** | ***p* of**  **random**  **sampling** | **FDR-**  **adjusted**  ***p*** |
| *ADAM5P* | 1 | 0.00034187 | 0.00114192 | *APC2* | 1 | 0.00030599 | 0.00112327 | *AGPAT2* | 1 | 0.00013928 | 0.00044842 |
| *B3GNT3* | 1 | 0.00034187 | 0.00114192 | *ASAP2* | 1 | 0.00030599 | 0.00112327 | *ANKFN1* | 1 | 0.00013928 | 0.00044842 |
| *BICD2* | 1 | 0.00034187 | 0.00114192 | *BMP6* | 1 | 0.00030599 | 0.00112327 | *CELSR2* | 1 | 0.00013928 | 0.00044842 |
| *C1orf35* | 1 | 0.00034187 | 0.00114192 | *C21orf96* | 1 | 0.00030599 | 0.00112327 | *CHST11* | 1 | 0.00013928 | 0.00044842 |
| *CDH15* | 1 | 0.00034187 | 0.00114192 | *CCDC138* | 1 | 0.00030599 | 0.00112327 | *D2HGDH* | 1 | 0.00013928 | 0.00044842 |
| *DPPA4* | 1 | 0.00034187 | 0.00114192 | *DENND3* | 1 | 0.00030599 | 0.00112327 | *FAM138B* | 1 | 0.00013928 | 0.00044842 |
| *GLIPR1L2* | 1 | 0.00034187 | 0.00114192 | *DLEU2L* | 1 | 0.00030599 | 0.00112327 | *FOXK1* | 1 | 0.00013928 | 0.00044842 |
| *HBM* | 1 | 0.00034187 | 0.00114192 | *FOXF1* | 1 | 0.00030599 | 0.00112327 | *KIF13B* | 1 | 0.00013928 | 0.00044842 |
| *HLA-DQB2* | 1 | 0.00034187 | 0.00114192 | *GRIK3* | 1 | 0.00030599 | 0.00112327 | *KSR1* | 1 | 0.00013928 | 0.00044842 |
| *HRNBP3* | 1 | 0.00034187 | 0.00114192 | *LOC254559* | 1 | 0.00030599 | 0.00112327 | *PPA2* | 1 | 0.00013928 | 0.00044842 |
| *IRS1* | 1 | 0.00034187 | 0.00114192 | *LOC728606* | 1 | 0.00030599 | 0.00112327 | *QPRT* | 1 | 0.00013928 | 0.00044842 |
| *ITFG3* | 1 | 0.00034187 | 0.00114192 | *MAPK11* | 1 | 0.00030599 | 0.00112327 | *RIMS4* | 1 | 0.00013928 | 0.00044842 |
| *LOC100101938* | 1 | 0.00034187 | 0.00114192 | *MT1L* | 1 | 0.00030599 | 0.00112327 | *UCK2* | 1 | 0.00013928 | 0.00044842 |
| *LOC149837* | 1 | 0.00034187 | 0.00114192 | *NCLN* | 1 | 0.00030599 | 0.00112327 | *VNN3* | 1 | 0.00013928 | 0.00044842 |
| *LOC619207* | 1 | 0.00034187 | 0.00114192 | *NECAB2* | 1 | 0.00030599 | 0.00112327 | *ASCL2* | 2 | 0.00027856 | 0.00044842 |
| *LOC650226* | 1 | 0.00034187 | 0.00114192 | *OR2W1* | 1 | 0.00030599 | 0.00112327 | *BAI2* | 2 | 0.00027856 | 0.00044842 |
| *LPHN1* | 1 | 0.00034187 | 0.00114192 | *OR4C11* | 1 | 0.00030599 | 0.00112327 | *CCT6P1* | 2 | 0.00027856 | 0.00044842 |
| *LRIT2* | 1 | 0.00034187 | 0.00114192 | *PAPLN* | 1 | 0.00030599 | 0.00112327 | *CHKA* | 2 | 0.00027856 | 0.00044842 |
| *MIR193A* | 1 | 0.00034187 | 0.00114192 | *RXRA* | 1 | 0.00030599 | 0.00112327 | *CXCR1* | 2 | 0.00027856 | 0.00044842 |
| *MMP9* | 1 | 0.00034187 | 0.00114192 | *RYBP* | 1 | 0.00030599 | 0.00112327 | *DCAF5* | 2 | 0.00027856 | 0.00044842 |
| *PLCH2* | 1 | 0.00034187 | 0.00114192 | *TMEM84* | 1 | 0.00030599 | 0.00112327 | *GMDS* | 2 | 0.00027856 | 0.00044842 |
| *PRKCG* | 1 | 0.00034187 | 0.00114192 | *TNFRSF9* | 1 | 0.00030599 | 0.00112327 | *GPR37* | 2 | 0.00027856 | 0.00044842 |
| *SLC19A1* | 1 | 0.00034187 | 0.00114192 | *TRIO* | 1 | 0.00030599 | 0.00112327 | *KDM6B* | 2 | 0.00027856 | 0.00044842 |
| *SPRN* | 1 | 0.00034187 | 0.00114192 | *UNC93B1* | 1 | 0.00030599 | 0.00112327 | *KIAA1804* | 2 | 0.00027856 | 0.00044842 |
| *TNFAIP8L3* | 1 | 0.00034187 | 0.00114192 | *UNCX* | 1 | 0.00030599 | 0.00112327 | *LHFPL2* | 2 | 0.00027856 | 0.00044842 |
| *TRIM31* | 1 | 0.00034187 | 0.00114192 | *VPS37D* | 1 | 0.00030599 | 0.00112327 | *LSG1* | 2 | 0.00027856 | 0.00044842 |
| *TRPM8* | 1 | 0.00034187 | 0.00114192 | *ZNF517* | 1 | 0.00030599 | 0.00112327 | *LTC4S* | 2 | 0.00027856 | 0.00044842 |
| *USP29* | 1 | 0.00034187 | 0.00114192 | *ANKRD34C* | 2 | 0.00061199 | 0.00112327 | *MSH6* | 2 | 0.00027856 | 0.00044842 |
| *VPS54* | 1 | 0.00034187 | 0.00114192 | *ARAP2* | 2 | 0.00061199 | 0.00112327 | *OSBPL5* | 2 | 0.00027856 | 0.00044842 |
| *ABCA6* | 2 | 0.00068374 | 0.00114192 | *BCL11B* | 2 | 0.00061199 | 0.00112327 | *PCGF3* | 2 | 0.00027856 | 0.00044842 |
| *ACADL* | 2 | 0.00068374 | 0.00114192 | *C17orf64* | 2 | 0.00061199 | 0.00112327 | *PELI2* | 2 | 0.00027856 | 0.00044842 |
| *ANKFY1* | 2 | 0.00068374 | 0.00114192 | *C20orf195* | 2 | 0.00061199 | 0.00112327 | *PEX10* | 2 | 0.00027856 | 0.00044842 |
| *ANO4* | 2 | 0.00068374 | 0.00114192 | *C3orf50* | 2 | 0.00061199 | 0.00112327 | *POU2F1* | 2 | 0.00027856 | 0.00044842 |
| *ATG4C* | 2 | 0.00068374 | 0.00114192 | *C5orf30* | 2 | 0.00061199 | 0.00112327 | *RPN1* | 2 | 0.00027856 | 0.00044842 |
| *ATP6V1C1* | 2 | 0.00068374 | 0.00114192 | *CACNA1C* | 2 | 0.00061199 | 0.00112327 | *SAMD4A* | 2 | 0.00027856 | 0.00044842 |
| *BAI1* | 2 | 0.00068374 | 0.00114192 | *CASS4* | 2 | 0.00061199 | 0.00112327 | *SGPL1* | 2 | 0.00027856 | 0.00044842 |
| *C11orf87* | 2 | 0.00068374 | 0.00114192 | *CATSPERG* | 2 | 0.00061199 | 0.00112327 | *SH3PXD2A* | 2 | 0.00027856 | 0.00044842 |
| *CCDC11* | 2 | 0.00068374 | 0.00114192 | *CDKAL1* | 2 | 0.00061199 | 0.00112327 | *TBX4* | 2 | 0.00027856 | 0.00044842 |
| *CCDC144NL* | 2 | 0.00068374 | 0.00114192 | *CDKN1C* | 2 | 0.00061199 | 0.00112327 | *TDRD3* | 2 | 0.00027856 | 0.00044842 |
| *CCDC67* | 2 | 0.00068374 | 0.00114192 | *CLIC4* | 2 | 0.00061199 | 0.00112327 | *TMEM88* | 2 | 0.00027856 | 0.00044842 |
| *CDK2AP1* | 2 | 0.00068374 | 0.00114192 | *DHX37* | 2 | 0.00061199 | 0.00112327 | *TNFAIP6* | 2 | 0.00027856 | 0.00044842 |
| *CHST9* | 2 | 0.00068374 | 0.00114192 | *DYRK2* | 2 | 0.00061199 | 0.00112327 | *AFF1* | 3 | 0.00041784 | 0.00057453 |
| *CHUK* | 2 | 0.00068374 | 0.00114192 | *ELOF1* | 2 | 0.00061199 | 0.00112327 | *BCL11A* | 3 | 0.00041784 | 0.00057453 |
| *CLCN7* | 2 | 0.00068374 | 0.00114192 | *FAM160A1* | 2 | 0.00061199 | 0.00112327 | *GGA1* | 3 | 0.00041784 | 0.00057453 |
| *CLDN11* | 2 | 0.00068374 | 0.00114192 | *FANCC* | 2 | 0.00061199 | 0.00112327 | *OSCP1* | 3 | 0.00041784 | 0.00057453 |
| *COX6A2* | 2 | 0.00068374 | 0.00114192 | *FLNB* | 2 | 0.00061199 | 0.00112327 | *SETD4* | 3 | 0.00041784 | 0.00057453 |
| *CSTA* | 2 | 0.00068374 | 0.00114192 | *GLRA1* | 2 | 0.00061199 | 0.00112327 | *SPEF2* | 3 | 0.00041784 | 0.00057453 |
| *CUEDC2* | 2 | 0.00068374 | 0.00114192 | *GNB1* | 2 | 0.00061199 | 0.00112327 | *TFRC* | 3 | 0.00041784 | 0.00057453 |
| *CYP4V2* | 2 | 0.00068374 | 0.00114192 | *GYPC* | 2 | 0.00061199 | 0.00112327 | *BDH1* | 4 | 0.00055712 | 0.00069377 |
| *D4S234E* | 2 | 0.00068374 | 0.00114192 | *HCN4* | 2 | 0.00061199 | 0.00112327 | *ETS1* | 4 | 0.00055712 | 0.00069377 |
| *DLK2* | 2 | 0.00068374 | 0.00114192 | *IRS2* | 2 | 0.00061199 | 0.00112327 | *HDAC4* | 4 | 0.00055712 | 0.00069377 |
| *ELAVL3* | 2 | 0.00068374 | 0.00114192 | *KCNA4* | 2 | 0.00061199 | 0.00112327 | *NSMCE2* | 4 | 0.00055712 | 0.00069377 |
| *EMILIN2* | 2 | 0.00068374 | 0.00114192 | *KHDC1* | 2 | 0.00061199 | 0.00112327 | *ZNF438* | 4 | 0.00055712 | 0.00069377 |
| *FGF18* | 2 | 0.00068374 | 0.00114192 | *LAIR2* | 2 | 0.00061199 | 0.00112327 | *DDX28* | 5 | 0.0006964 | 0.00075348 |
| *FILIP1* | 2 | 0.00068374 | 0.00114192 | *LARP1B* | 2 | 0.00061199 | 0.00112327 | *DUS2L* | 5 | 0.0006964 | 0.00075348 |
| *FN3K* | 2 | 0.00068374 | 0.00114192 | *LMNB2* | 2 | 0.00061199 | 0.00112327 | *ERCC8* | 5 | 0.0006964 | 0.00075348 |
| *FNDC1* | 2 | 0.00068374 | 0.00114192 | *LRRC28* | 2 | 0.00061199 | 0.00112327 | *HOXA11* | 5 | 0.0006964 | 0.00075348 |
| *GRPEL1* | 2 | 0.00068374 | 0.00114192 | *MIR1250* | 2 | 0.00061199 | 0.00112327 | *HSPBAP1* | 5 | 0.0006964 | 0.00075348 |
| *HLA-DMB* | 2 | 0.00068374 | 0.00114192 | *NOV* | 2 | 0.00061199 | 0.00112327 | *NDUFAF2* | 5 | 0.0006964 | 0.00075348 |
| *HOXA13* | 2 | 0.00068374 | 0.00114192 | *NUP43* | 2 | 0.00061199 | 0.00112327 | *NEBL* | 5 | 0.0006964 | 0.00075348 |
| *HTR1D* | 2 | 0.00068374 | 0.00114192 | *OTUB1* | 2 | 0.00061199 | 0.00112327 | *TRMT6* | 5 | 0.0006964 | 0.00075348 |
| *IFI44L* | 2 | 0.00068374 | 0.00114192 | *PCDH9* | 2 | 0.00061199 | 0.00112327 | *P2RY12* | 6 | 0.00083568 | 0.0008896 |
| *ING3* | 2 | 0.00068374 | 0.00114192 | *PCYOX1* | 2 | 0.00061199 | 0.00112327 | *ARFIP1* | 7 | 0.00097496 | 0.00098996 |
| *KIF25* | 2 | 0.00068374 | 0.00114192 | *PNPLA8* | 2 | 0.00061199 | 0.00112327 | *CPSF3* | 7 | 0.00097496 | 0.00098996 |
| *MANBA* | 2 | 0.00068374 | 0.00114192 | *RAP1GDS1* | 2 | 0.00061199 | 0.00112327 | *MBD4* | 7 | 0.00097496 | 0.00098996 |
| *MGRN1* | 2 | 0.00068374 | 0.00114192 | *SERPINB13* | 2 | 0.00061199 | 0.00112327 | *MED12L* | 19 | 0.00264633 | 0.00264633 |
| *MKRN2* | 2 | 0.00068374 | 0.00114192 | *SRRT* | 2 | 0.00061199 | 0.00112327 |  |  |  |  |
| *MOG* | 2 | 0.00068374 | 0.00114192 | *ST8SIA2* | 2 | 0.00061199 | 0.00112327 |  |  |  |  |
| *NKTR* | 2 | 0.00068374 | 0.00114192 | *STK39* | 2 | 0.00061199 | 0.00112327 |  |  |  |  |
| *NLGN1* | 2 | 0.00068374 | 0.00114192 | *TNKS2* | 2 | 0.00061199 | 0.00112327 |  |  |  |  |
| *PACSIN1* | 2 | 0.00068374 | 0.00114192 | *TPX2* | 2 | 0.00061199 | 0.00112327 |  |  |  |  |
| *PCDHB3* | 2 | 0.00068374 | 0.00114192 | *UBE2H* | 2 | 0.00061199 | 0.00112327 |  |  |  |  |
| *PCGF3* | 2 | 0.00068374 | 0.00114192 | *UTP23* | 2 | 0.00061199 | 0.00112327 |  |  |  |  |
| *PI16* | 2 | 0.00068374 | 0.00114192 | *VASH1* | 2 | 0.00061199 | 0.00112327 |  |  |  |  |
| *POU5F1B* | 2 | 0.00068374 | 0.00114192 | *VSTM2A* | 2 | 0.00061199 | 0.00112327 |  |  |  |  |
| *RNF103* | 2 | 0.00068374 | 0.00114192 | *ZNF57* | 2 | 0.00061199 | 0.00112327 |  |  |  |  |
| *ROBO3* | 2 | 0.00068374 | 0.00114192 | *ZNF605* | 2 | 0.00061199 | 0.00112327 |  |  |  |  |
| *RPL24* | 2 | 0.00068374 | 0.00114192 | *ANP32E* | 3 | 0.00091798 | 0.00129231 |  |  |  |  |
| *RSPO2* | 2 | 0.00068374 | 0.00114192 | *CHTF18* | 3 | 0.00091798 | 0.00129231 |  |  |  |  |
| *SGSM1* | 2 | 0.00068374 | 0.00114192 | *CNP* | 3 | 0.00091798 | 0.00129231 |  |  |  |  |
| *SHROOM1* | 2 | 0.00068374 | 0.00114192 | *DEAF1* | 3 | 0.00091798 | 0.00129231 |  |  |  |  |
| *SLC6A3* | 2 | 0.00068374 | 0.00114192 | *DNMBP* | 3 | 0.00091798 | 0.00129231 |  |  |  |  |
| *SNRPC* | 2 | 0.00068374 | 0.00114192 | *FASTK* | 3 | 0.00091798 | 0.00129231 |  |  |  |  |
| *STMN2* | 2 | 0.00068374 | 0.00114192 | *HOXB4* | 3 | 0.00091798 | 0.00129231 |  |  |  |  |
| *SVOPL* | 2 | 0.00068374 | 0.00114192 | *LARP1* | 3 | 0.00091798 | 0.00129231 |  |  |  |  |
| *TDH* | 2 | 0.00068374 | 0.00114192 | *LCK* | 3 | 0.00091798 | 0.00129231 |  |  |  |  |
| *TEKT1* | 2 | 0.00068374 | 0.00114192 | *LSR* | 3 | 0.00091798 | 0.00129231 |  |  |  |  |
| *TMEM182* | 2 | 0.00068374 | 0.00114192 | *MYT1L* | 3 | 0.00091798 | 0.00129231 |  |  |  |  |
| *TMEM233* | 2 | 0.00068374 | 0.00114192 | *PDLIM2* | 3 | 0.00091798 | 0.00129231 |  |  |  |  |
| *TMIE* | 2 | 0.00068374 | 0.00114192 | *PPP1R9A* | 3 | 0.00091798 | 0.00129231 |  |  |  |  |
| *TRIM42* | 2 | 0.00068374 | 0.00114192 | *PPP2R5A* | 3 | 0.00091798 | 0.00129231 |  |  |  |  |
| *UPF2* | 2 | 0.00068374 | 0.00114192 | *PSPC1* | 3 | 0.00091798 | 0.00129231 |  |  |  |  |
| *VWA3B* | 2 | 0.00068374 | 0.00114192 | *RPL28* | 3 | 0.00091798 | 0.00129231 |  |  |  |  |
| *WNK4* | 2 | 0.00068374 | 0.00114192 | *SAMD11* | 3 | 0.00091798 | 0.00129231 |  |  |  |  |
| *ZFYVE28* | 2 | 0.00068374 | 0.00114192 | *SPHK2* | 3 | 0.00091798 | 0.00129231 |  |  |  |  |
| *ZNF208* | 2 | 0.00068374 | 0.00114192 | *SYNPO2L* | 3 | 0.00091798 | 0.00129231 |  |  |  |  |
| *ZNF423* | 2 | 0.00068374 | 0.00114192 | *TRAPPC9* | 3 | 0.00091798 | 0.00129231 |  |  |  |  |
| *C11orf49* | 3 | 0.00102561 | 0.00149684 | *TTC23* | 3 | 0.00091798 | 0.00129231 |  |  |  |  |
| *CBFA2T3* | 3 | 0.00102561 | 0.00149684 | *USF2* | 3 | 0.00091798 | 0.00129231 |  |  |  |  |
| *FAM160B1* | 3 | 0.00102561 | 0.00149684 | *USP19* | 3 | 0.00091798 | 0.00129231 |  |  |  |  |
| *FBXW2* | 3 | 0.00102561 | 0.00149684 | *ZFHX3* | 3 | 0.00091798 | 0.00129231 |  |  |  |  |
| *IFT172* | 3 | 0.00102561 | 0.00149684 | *BMP8A* | 4 | 0.00122398 | 0.00158462 |  |  |  |  |
| *KCNMA1* | 3 | 0.00102561 | 0.00149684 | *DIP2C* | 4 | 0.00122398 | 0.00158462 |  |  |  |  |
| *LOC100128164* | 3 | 0.00102561 | 0.00149684 | *FGFRL1* | 4 | 0.00122398 | 0.00158462 |  |  |  |  |
| *LOC402377* | 3 | 0.00102561 | 0.00149684 | *FLJ35024* | 4 | 0.00122398 | 0.00158462 |  |  |  |  |
| *MAB21L1* | 3 | 0.00102561 | 0.00149684 | *GCNT2* | 4 | 0.00122398 | 0.00158462 |  |  |  |  |
| *PBX3* | 3 | 0.00102561 | 0.00149684 | *RASGRF2* | 4 | 0.00122398 | 0.00158462 |  |  |  |  |
| *RGS12* | 3 | 0.00102561 | 0.00149684 | *RPSAP52* | 4 | 0.00122398 | 0.00158462 |  |  |  |  |
| *SEC62* | 3 | 0.00102561 | 0.00149684 | *RUFY1* | 4 | 0.00122398 | 0.00158462 |  |  |  |  |
| *SYCE1* | 3 | 0.00102561 | 0.00149684 | *VLDLR* | 4 | 0.00122398 | 0.00158462 |  |  |  |  |
| *YOD1* | 3 | 0.00102561 | 0.00149684 | *ANLN* | 5 | 0.00152997 | 0.00180363 |  |  |  |  |
| *ABCF1* | 4 | 0.00136748 | 0.00183084 | *C10orf118* | 5 | 0.00152997 | 0.00180363 |  |  |  |  |
| *ANKRD2* | 4 | 0.00136748 | 0.00183084 | *CLCF1* | 5 | 0.00152997 | 0.00180363 |  |  |  |  |
| *HAND2* | 4 | 0.00136748 | 0.00183084 | *DNAI1* | 5 | 0.00152997 | 0.00180363 |  |  |  |  |
| *LOC286002* | 4 | 0.00136748 | 0.00183084 | *EFCAB7* | 5 | 0.00152997 | 0.00180363 |  |  |  |  |
| *LTBP3* | 4 | 0.00136748 | 0.00183084 | *EMX2* | 5 | 0.00152997 | 0.00180363 |  |  |  |  |
| *NEU1* | 4 | 0.00136748 | 0.00183084 | *EMX2OS* | 5 | 0.00152997 | 0.00180363 |  |  |  |  |
| *NPSR1* | 4 | 0.00136748 | 0.00183084 | *HMGA2* | 5 | 0.00152997 | 0.00180363 |  |  |  |  |
| *SLC26A4* | 4 | 0.00136748 | 0.00183084 | *IFT140* | 5 | 0.00152997 | 0.00180363 |  |  |  |  |
| *TNFRSF6B* | 4 | 0.00136748 | 0.00183084 | *RTN4* | 5 | 0.00152997 | 0.00180363 |  |  |  |  |
| *ZDHHC24* | 4 | 0.00136748 | 0.00183084 | *RUNX1* | 5 | 0.00152997 | 0.00180363 |  |  |  |  |
| *LIN7A* | 5 | 0.00170935 | 0.00211385 | *C5orf38* | 6 | 0.00183597 | 0.00211282 |  |  |  |  |
| *MOBKL2A* | 5 | 0.00170935 | 0.00211385 | *C9orf25* | 6 | 0.00183597 | 0.00211282 |  |  |  |  |
| *NTM* | 5 | 0.00170935 | 0.00211385 | *THAP2* | 6 | 0.00183597 | 0.00211282 |  |  |  |  |
| *PTPRN2* | 5 | 0.00170935 | 0.00211385 | *C5orf13* | 7 | 0.00214196 | 0.00233522 |  |  |  |  |
| *RARA* | 5 | 0.00170935 | 0.00211385 | *DNAJC7* | 7 | 0.00214196 | 0.00233522 |  |  |  |  |
| *RIBC2* | 5 | 0.00170935 | 0.00211385 | *IGF2AS* | 7 | 0.00214196 | 0.00233522 |  |  |  |  |
| *SMC1B* | 5 | 0.00170935 | 0.00211385 | *JMJD1C* | 7 | 0.00214196 | 0.00233522 |  |  |  |  |
| *SORCS2* | 5 | 0.00170935 | 0.00211385 | *KIAA0895* | 7 | 0.00214196 | 0.00233522 |  |  |  |  |
| *TRIM23* | 5 | 0.00170935 | 0.00211385 | *SND1* | 7 | 0.00214196 | 0.00233522 |  |  |  |  |
| *ZNF490* | 5 | 0.00170935 | 0.00211385 | *PTCD3* | 8 | 0.00244796 | 0.00264891 |  |  |  |  |
| *ART3* | 6 | 0.00205122 | 0.00247983 | *AATK* | 9 | 0.00275395 | 0.0029362 |  |  |  |  |
| *C5orf44* | 6 | 0.00205122 | 0.00247983 | *MIR548H4* | 9 | 0.00275395 | 0.0029362 |  |  |  |  |
| *NBEA* | 6 | 0.00205122 | 0.00247983 | *LOC100130987* | 10 | 0.00305995 | 0.00323863 |  |  |  |  |
| *C20orf26* | 7 | 0.00239309 | 0.00285059 | *IGF2* | 14 | 0.00428393 | 0.00450123 |  |  |  |  |
| *MIR548F5* | 7 | 0.00239309 | 0.00285059 | *INS-IGF2* | 18 | 0.00550791 | 0.00574566 |  |  |  |  |
| *PCDHA11* | 25 | 0.00854676 | 0.01010638 | *C21orf29* | 35 | 0.01070982 | 0.01109232 |  |  |  |  |
| *PCDHA10* | 31 | 0.01059798 | 0.0124411 | *PCDHA5* | 43 | 0.01315778 | 0.01353105 |  |  |  |  |
| *PCDHA9* | 33 | 0.01128172 | 0.01314848 | *PCDHA4* | 47 | 0.01438176 | 0.0146856 |  |  |  |  |
| *PCDHA8* | 35 | 0.01196546 | 0.01384574 | *PCDHA3* | 51 | 0.01560574 | 0.01582401 |  |  |  |  |
| *PCDHGB6* | 36 | 0.01230733 | 0.01414034 | *PCDHA2* | 55 | 0.01682972 | 0.0169466 |  |  |  |  |
| *PCDHA7* | 38 | 0.01299107 | 0.0148208 | *PCDHA1* | 57 | 0.01744171 | 0.01744171 |  |  |  |  |
| *PCDHGA9* | 39 | 0.01333294 | 0.01510445 |  |  |  |  |  |  |  |  |
| *PCDHA6* | 41 | 0.01401668 | 0.01576876 |  |  |  |  |  |  |  |  |
| *PCDHGB5* | 42 | 0.01435855 | 0.01604197 |  |  |  |  |  |  |  |  |
| *PCDHA5* | 43 | 0.01470042 | 0.01631143 |  |  |  |  |  |  |  |  |
| *PCDHGA8* | 45 | 0.01538416 | 0.01695397 |  |  |  |  |  |  |  |  |
| *PCDHA4* | 47 | 0.0160679 | 0.01758784 |  |  |  |  |  |  |  |  |
| *PCDHGB4* | 48 | 0.01640977 | 0.0178415 |  |  |  |  |  |  |  |  |
| *PCDHA3* | 51 | 0.01743538 | 0.01870551 |  |  |  |  |  |  |  |  |
| *PCDHGA7* | 51 | 0.01743538 | 0.01870551 |  |  |  |  |  |  |  |  |
| *PCDHGA6* | 54 | 0.01846099 | 0.01967553 |  |  |  |  |  |  |  |  |
| *PCDHA2* | 55 | 0.01880286 | 0.01990891 |  |  |  |  |  |  |  |  |
| *PCDHGB3* | 56 | 0.01914473 | 0.02013927 |  |  |  |  |  |  |  |  |
| *PCDHA1* | 57 | 0.0194866 | 0.02036664 |  |  |  |  |  |  |  |  |
| *PCDHGA5* | 59 | 0.02017034 | 0.02094613 |  |  |  |  |  |  |  |  |
| *PCDHGB2* | 62 | 0.02119595 | 0.02187099 |  |  |  |  |  |  |  |  |
| *PCDHGA4* | 66 | 0.02256344 | 0.02313466 |  |  |  |  |  |  |  |  |
| *PCDHGB1* | 69 | 0.02358905 | 0.02403412 |  |  |  |  |  |  |  |  |
| *PCDHGA3* | 71 | 0.02427279 | 0.0245762 |  |  |  |  |  |  |  |  |
| *PCDHGA2* | 74 | 0.0252984 | 0.02545553 |  |  |  |  |  |  |  |  |
| *PCDHGA1* | 76 | 0.02598214 | 0.02598214 |  |  |  |  |  |  |  |  |
